# A Novel AtKEA Gene Family, Homolog of Bacterial K^+^/H^+^ Antiporters, Plays Potential Roles in K^+^ Homeostasis and Osmotic Adjustment in Arabidopsis

**DOI:** 10.1371/journal.pone.0081463

**Published:** 2013-11-20

**Authors:** Sheng Zheng, Ting Pan, Ligang Fan, Quan-Sheng Qiu

**Affiliations:** MOE Key Laboratory of Cell Activities and Stress Adaptations, School of Life Sciences, Lanzhou University, Lanzhou, Gansu, China; Universität für Bodenkultur Wien (BOKU), Austria

## Abstract

AtKEAs, homologs of bacterial KefB/KefC, are predicted to encode K^+^/H^+^ antiporters in Arabidopsis. The *AtKEA* family contains six genes forming two subgroups in the cladogram: *AtKEA1-3* and *AtKEA4-6*. AtKEA1 and AtKEA2 have a long N-terminal domain; the full-length AtKEA1 was inactive in yeast. The transport activity was analyzed by expressing the *AtKEA* genes in yeast mutants lacking multiple ion carriers. AtKEAs conferred resistance to high K^+^ and hygromycin B but not to salt and Li^+^ stress. *AtKEAs* expressed in both the shoot and root of Arabidopsis. The expression of *AtKEA1*, *-3* and *-4* was enhanced under low K^+^ stress, whereas *AtKEA2* and *AtKEA5* were induced by sorbitol and ABA treatments. However, osmotic induction of *AtKEA2* and *AtKEA5* was not observed in *aba2-3* mutants, suggesting an ABA regulated mechanism for their osmotic response. *AtKEAs*’ expression may not be regulated by the SOS pathway since their expression was not affected in *sos* mutants. The GFP tagging analysis showed that AtKEAs distributed diversely in yeast. The Golgi localization of AtKEA3 was demonstrated by both the stably transformed seedlings and the transient expression in protoplasts. Overall, AtKEAs expressed and localized diversely, and may play roles in K^+^ homeostasis and osmotic adjustment in Arabidopsis.

## Introduction

Na^+^,K^+^/H^+^ antiporters are secondary transporters that exist in all kinds of life including bacteria, yeast, plants and animals [1], [2], [3], [4]. They are H^+^-coupled cotransporters whose biochemical activity is to transfer the Na^+^ or K^+^ across a membrane in exchange for protons (H^+^). Na^+^,K^+^/H^+^ antiporters form a large gene family, and currently there are more than 200 genes that have been annotated as Na^+^,K^+^/H^+^ antiporters [3]. The Na^+^,K^+^/H^+^ antiporter is categorized into the monovalent cation proton antiporter (CPA) gene family [5]. Na^+^,K^+^/H^+^ antiporters are critical for ion homeostasis and pH regulation in cells, and function in diverse cellular processes, including Na^+^ and K^+^ movement, salt tolerance, regulation of cell cycle and cell proliferation, vesicle trafficking and fusion, and biogenesis [6], [7], [8].

In the Arabidopsis genome, there are approximately 44 genes that are predicted to encode Na^+^,K^+^/H^+^ antiporters, including 8 *AtNHXs*, 28 *AtCHXs* and 6 *AtKEAs*. The *AtNHXs* belong to the CPA1 gene family while the *AtCHXs* and the *AtKEAs* are members of the CPA2 family [3], [4], [9], [10]. 

The function, transport activity, and regulatory mechanism of AtNHX transporters have been studied extensively [1], [2], [7], [11], [12]. AtNHXs are involved in the regulation of cellular ion and pH homeostasis, and play an important role in salt tolerance, K^+^ homeostasis, vesicle trafficking, and plant growth and development [8], [11], [12], [13]. Overexpression of *AtNHX1* and *SOS1/AtNHX7* reduces cytoplasmic Na^+^ content and enhances salt tolerance in Arabidopsis [14], [15], [16]. SOS1 activity is regulated by SOS2 kinase [17], [18], [19]. SOS1 is activated by the removal of a C-terminal auto-inhibitory domain upon phosphorylation by the SOS2/SOS3 complex [20]. AtNHX1 may also be regulated by SOS2 kinase [21]. CaM binds and inhibits the Na^+^/H^+^ antiport activity of AtNHX1 [22]. AtNHX1 and LeNHX2 have a K^+^/H^+^ transport activity and mediate K^+^ compartmentation in vacuoles [23], [24], [25], [26], [27]. The *NHX* genes in *Ipomea tricolor* and *Ipomea Nil* are involved in vacuolar pH regulation; mutation of a *NHX* gene in *Ipomea Nil* abolished the colour change in flowers, a process that is controlled by an increase in vacuolar pH [28], [29]. *nhx1nhx2* double knockout mutants showed significantly reduced growth, abnormal stamens and lacked silique formation, indicating that AtNHX1 and AtNHX2 function in cell expansion and flower development [30]. *nhx1nhx2* double mutants had reduced ability in creating the vacuolar K^+^ pool, impaired osmoregulation, and compromised turgor generation for cell expansion, indicating that AtNHX1 and AtNHX2 are essential for active K^+^ uptake at the tonoplast, turgor regulation, and stomatal function [31]. AtNHX5 and AtNHX6 play an important role in endosomal sorting and stress responses. The *nhx5nhx6* double knockout mutants had reduced growth and increased sensitivity to salinity. Vacuolar trafficking was affected in *nhx5nhx6* [32]. 

The function and expression of *AtCHX* genes are beginning to be explored. AtCHXs regulate K^+^ and pH homeostasis, and function in controlling membrane trafficking, osmoregulation, and pollen growth and development [4], [10], [33], [34]. Most of the *AtCHX* genes are preferentially expressed in the male gametophyte and sporophytic tissues, suggesting roles in pollen desiccation at maturation and rehydration on germination [10]. AtCHX13 is localized to the plasma membrane K^+^ transporter which is responsible for K^+^ uptake in roots [35]. AtCHX17 is mainly expressed in roots and mediates K^+^ homeostasis [36]. AtCHX20 is highly expressed in guard cells, and functions in osmoregulation of stomatal opening through K^+^ movement and pH regulation in guard cells [37]. AtCHX21 is a putative Na^+^ transporter regulating Na^+^ homehostasis in xylem and Na^+^ accumulation in leaves [38]. AtCHX23 functions in the adjustment of pH in the cytosol and possibly in maintaining a high pH level in the chloroplast stroma [39]. In the *Atchx21chx23* double mutant, pollen tubes failed to target ovules which resulted in impaired pollen fertility, indicating a role in pollen tube guidance [34]. AtCHX17 and AtCHX21 have K^+^ transport activities, and are involved in protein sorting [33]. AtCHX20 might be a K^+^/H^+^ symporter and AtCHX17 might function as a K^+^ channel [33].

AtKEAs are homologs of EcKefB and EcKefC, K^+^ efflux transporters in *E. Coli* [4].. EcKefB/EcKefC is activated by adducts of glutathione and negatively regulated by glutathione, and function in survival of stress caused by toxic metabolites. The C-terminus of EcKefB/EcKefC has a KTN domain, which is shared by many bacterial potassium channels and transporters, including EcKch, EcTrkA, EcYbal, EcKefB and EcKefC [40]. Binding of the ancillary protein EcKefF and GSH induces conformational changes in EcKefC KTN dimmers and activates the transport activity of EcKefB/EcKefC [41], [42], [43], [44]. EcKefC may function as a K^+^/H^+^ antiporter, although it was previously thought to act as a K^+^ channel [45]. However, the function of the *AtKEA* gene family remains largely uncharacterized. So far, only AtKEA2 has been characterized experimentally [46]. AtKEA2 was targeted at chloroplasts and expressed highly in aerial parts of Arabidopsis. AtKEA2 conferred resistance to hygromycin B, high K^+^, and Na^+^ stress in *scnhx1* mutants. AtKEA2 was shown to have cation/H^+^ antiport activity when measured with reconstituted lipososmes. In another study, AtKEA1and AtKEA3 were detected from chloroplast preparations in *Arabidopsis* by mass spectrometry [47]. 

In this report, the function and expression of the *AtKEA* gene family have been studied. We have used yeast growth, RT-qPCR and GFP labeling techniques to characterize the transport activity, gene expression and cellular localization of the *AtKEA* family. Our results show that AtKEAs are diversely expressed and distributed in cells, and may function in facilitating K^+^ homeostasis and osmotic adjustment in Arabidopsis.

## Materials and Methods

### Plant materials and growth conditions

*Arabidopsis thaliana* ecotypes Columbia (Col-0), mutants, and transgenic lines were used in this study. *Arabidopsis* mutant *aba2-3* was ordered from ABRC；*sos1-1，sos2-1，sos3-1* were gifts from Dr. Jian-Kang Zhu. In the growth chamber, plants were grown on compost (Pindstrup Substrate, Latvia) and subirrigated with tap water. Greenhouse conditions were as follows: 16-h-light / 8-h-dark cycles, light intensity 100 μmol s^-1^ m^-2^ photosynthetically active radiation, temperature 22°C. For plate-grown plants, Arabidopsis thaliana seeds were surface sterilized with 20% (v/v) bleach, after cold treatment at 4°C for 3 days, the seeds were germinated on plates with 1/2 strength Murashige and Skoog (MS) medium containing 0.8% agar, pH 5.8. For growth at low potassium, seedlings were cultured on a modified MS medium containing various concentrations of KCl [48]. 

### Bioinformatic analysis

The predicted amino acid sequences of AtKEAs were collected from the GenBank (http://www.ncbi.nlm.nih.gov). Pairwise amino acid sequence were compared using EMBOSS Needle (http://www.ebi.ac.uk/Tools/psa/) [49]. Proteins were compared by multiple alignments using the ClustalX 2.1 method [50]. Phylogenetic analysis was conducted in MEGA 5.02 [51]. The alignment is based on the complete amino acid sequences. Evolutionary distances were computed by the Neighbor Joining method. Bootstrap analysis for each branch was performed 10,000 times. 

### Yeast Strains, media, and growth conditions

*Saccharomyces cerevisiae* strains W303-1B (MATα *leu2-13 112, ura3-1, trp1-1, his3-11 15, ade2-1, can1-100*), ANT3 (*ena1-4Δ::HIS3, nha1Δ::LEU2*), AXT3 (*ena1-4Δ::HIS3, nha1Δ::LEU2, nhx1Δ*:*: TRP1*) and AXT4K (*ena1-4Δ::HIS3, nha1Δ::LEU2, nhx1Δ*:*: TRP1, kha1Δ::KanMX6*) were gifts from Dr. Jose M. Pardo [52], [53], [54]. All strains used were derivatives of W303-1B. Untransformed strains were grown at 30°C in YPD medium (1% yeast extract, 2% peptone and 2% glucose). Transformation of yeast cells was performed by the lithium acetate method. After transformation, strains were grown on selective Hartwell's complete (SC) medium or APG medium (10mM arginine, 8mM phosphoric acid, 2 mM MgSO_4_, 1 mM KCl, 0.2 mM CaCl_2_, 2% glucose, and trace minerals and vitamins). NaCl, KCl, or hygromycin B was added to the medium. Drop test media contained 20 mM MES, and pH was adjusted to 7.5 with arginine [33] or to acidic pH values with phosphoric acid [55]. 

### Functional expression of AtKEAs in yeast

To clone the full length CDS of AtKEA1, we separated AtKEA1 into two segments, AtKEA1-F1870 and AtKEA1-L1712, respectively, by an EcoRI restriction site in the middle of the gene. Primers KEA1-SmaI F and KEA1-1996R (Table S1) were used to amplify the AtKEA1-F1870 using the PrimeSTAR^TM^ HS DNA Polymerase (TaKaRa) by PCR. AtKEA1-L1712 was amplified with primers KEA1-1862F and KEA1-XhoI R (Table S1) by PCR. The PCR product AtKEA1-F1870 was ligated into the yeast expression vector pDR196 at SmaI-EcoRI sites by T4 DNA ligase (promega), resulting in pDR196-AtKEA1-F1870. Then, the PCR product AtKEA1-L1712 was inserted into pDR196-AtKEA1-F1870 at EcoRI-XhoI sites to obtain the full length AtKEA1 (named as pDR196-AtKEA1). The full length CDS of AtKEA1 was verified by sequencing. 

For AtKEA2 (gene accession AT4G00630.1), a XhoI site in the middle of the gene was chosen to separate the gene into two pieces of A_1_-G_2123_ and A_2124_-A_3525_ (AtKEA2-F2123 and AtKEA2-L1402, respectively). Primers KEA2-NotI F and KEA2-2196R (Table S1) were used to amplify the AtKEA2-F2123 by PCR. AtKEA2-L1402 was amplified with primers KEA2-2052F and KEA2-XbaI R (Table S1) by PCR. The PCR product AtKEA2-F2123 was ligated into the yeast expression vector pYES2 at NotI-XhoI sites, resulting in pYES2- AtKEA2-F2123. Then, the PCR product AtKEA2-L1402 was inserted into the vector pYES2-AtKEA2-F2123 at XhoI-XbaI sites to obtain the full length AtKEA2 (named as pYES2-AtKEA2). However, after combining, mutations were generated at the region of A_1302_-A_1308_ which contains consecutive 7 adenines (As). Either 1 to 2 As were missing or added in the gene sequence. Similar mutations happened when we chose a KpnI site which separated the gene into two pieces of A_1_-C_1359_ and A_1360_-A_3525_. Thus, the full-length cDNA of AtKEA2 was not cloned in our experiments.

To clone AtsKEA1(short form of AtKEA1 with 1857bp nucleotides), AtKEA3, AtKEA4, AtNHX1 and AtCHX17, gene fragments were amplified by PCR from Arabidopsis cDNA (primers are listed in Table S1). To clone ScNHX1 and ScKHA1, gene fragments were amplified by PCR from the genomic DNA isolated from the *Saccharomyces cerevisiae* strain BJ3505(primers are listed in Table S1).The PCR fragments were cloned into the same sites of the plasmid pDR196. All gene fragments were verified by sequencing. 

The cDNAs of AtsKEA2 (short form of AtKEA2 with 1860bp nucleotides), AtKEA5 and AtKEA6, ordered from ABRC, were cloned in the yeast expression vector pDR196 with the promoter PMA1. 

All plasmids were transformed into the yeast strain AXT3 or AXT4K; the empty vector pDR196 was transformed into the same yeast strains as a control. For stress tolerance tests, yeast cells were normalized in water to A_600_ of 0.4. 4μl aliquots of each 10-fold serial dilution were spotted onto AP plates supplemented with KCl, or YPD plates supplemented with NaCl as indicated, and incubated at 30°C for 3 days. Resistance to hygromycin B was assayed in YPD medium.

### Quantitative real-time RT-PCR (RT-qPCR) analysis

14-day-old WT seedlings, growing on 1/2 MS plates, were transferred into the liquid 1/2 MS media without (control) or with 160 mM NaCl, 40 mM LiCl, 320 mM sorbitol or 100 μM ABA, respectively, and maintained for 8 h (Yokoi et al., 2002). The total RNA was isolated using the RNAiso Plus (TaKaRa). The first-strand cDNA was synthesized from the total RNA (1μg ) using the PrimeScript^®^ RT reagent kit with gDNA Eraser (TaKaRa), and was used as templates for PCR amplification. PCR amplification was performed with the CFX96 system (Bio-Rad) using the SYBR^®^ Premix Ex Taq™ (TaKaRa). The Arabidopsis Actin7 gene was used as an internal control, and differences in product levels among the tested samples during the linear amplification phase were used to calculate the differential gene expression [56]. The gene-specific primers are listed in Table S2. 

### Localization of the AtKEAs-GFP fusion proteins in yeast

To make GFP fusion constructs, we converted the vector pDR196 into a Gateway destination vector pDR196-GFP. GFP was fused at the C-terminal of plant or yeast proteins. Gene fragments of AtKEAs, AtNHX1 and AtCHX17 were amplified by PCR from Arabidopsis cDNAs (primers are listed in Table S3). To make GFP fusion constructs for ScNHX1 and ScKHA1, gene fragments were amplified by PCR from the genomic DNA isolated from the *Saccharomyces cerevisiae* strain BJ3505 (primers are listed in Table S3). PCR fragments were inserted into the plasmid pDR196-GFP using the Gateway technology (Invitrogen), respectively. Gene fragments were verified by sequencing.

The recombinant plasmids were transformed into the yeast strain W303-1B. Yeast cells grown to logarithmic phase at 30°C in SC-URA medium adjusted to pH 5.8. For FM4-64 staining, yeast cells grown exponentially were harvested and suspended in fresh YPD medium, and then were incubated with FM4-64 dye at a final concentration of 5μM. After incubation for 8h, the cells were washed four times with phosphate buffered saline (PBS) and concentrated by centrifugation. After mixing with 0.6% agarose, the cells were mounted on glass slides and observed by a confocal laser scanning microscope (FV1000, Olympus) [57]. 

### Localization of the RFP-AtKEA3 fusion protein in Arabidopsis protoplasts

Transient expression assay using protoplasts derived from the leaf mesophyll cells of Arabidopsis were performed as described [58]. RFP gene was fused in frame to AtKEA3 at its N-terminus. The AtKEA3 gene was amplified by using the following primers: 5′-AAAAAGCAGGCTTCATGGCAATTAGTACTATGTT-3′ and 5′-AGAAAGCTGGGTCTTAATCTTGAGCTTTATCAG -3′. The PCR fragment was inserted into the plasmid pUBN-RFP [59] using the Gateway technology (Invitrogen). Protoplasts were co-transfected with pUBN-RFP-AtKEA3 and a cis-Golgi marker GFP-AtSYP31. Arabidopsis seedlings of 4 weeks old were used for protoplast isolation. Fluorescence was visualized by a confocal laser scanning microscope (FV1000, Olympus).

### Localization of the AtKEA3-GFP fusion protein in stably transformed *Arabidopsis* seedlings

For the stable transformation assays with *Arabidopsis thaliana*, the GFP gene was fused in frame to AtKEA3 at its C-terminus. The AtKEA3 gene was amplified by using the following primers: 5′-AAAAAGCAGGCTTCATGGCAATTAGTACTATGTT-3′ and 5′-AGAAAGCTGGGTCATCTTGAGCTTTATCAGC-3′. The PCR fragment was inserted in plasmid pBIB-EGFP using the Gateway technology (Invitrogen), and the resulting construct was transformed into *Agrobacterium tumefaciens* GV3101. *Arabidopsis thaliana* (ecotype Columbia) wild-type plants were transformed [60]. The transgenic plants were screened by basta spray; the basta positive seedlings were re-confirmed with PCR amplification of the GFP fragment. GFP fluorescence was visualized under a confocal laser scanning microscope (FV1000, Olympus). The excitation wavelength for EGFP detection was 488 nm. The roots of the transgenic seedlings containing the 35S-AtKEA3-EGFP fusion protein were visualized under the confocal microscope.

The GenBank accession numbers for sequence data used in this article are: AtKEA1 (AEE27335); AtKEA2 (AEE81911); AtKEA3 (AEE82433); AtKEA4 (AEC06899); AtKEA5 (AED96117); AtKEA6 (AED91722); AtNHX1 (AAD16946); AtNHX2 (AAM08403); AtCHX1 (AEE29444); AtCHX17 (AEE84796); EcKefB (ACT40850); EcKefC (CAA40066); ScKHA1 (DAA08706); ScNHX1 (DAA12290).

## Results

### AtKEAs separate from AtNHXs and AtCHXs in the phylogenetic tree

**Figure 1:**
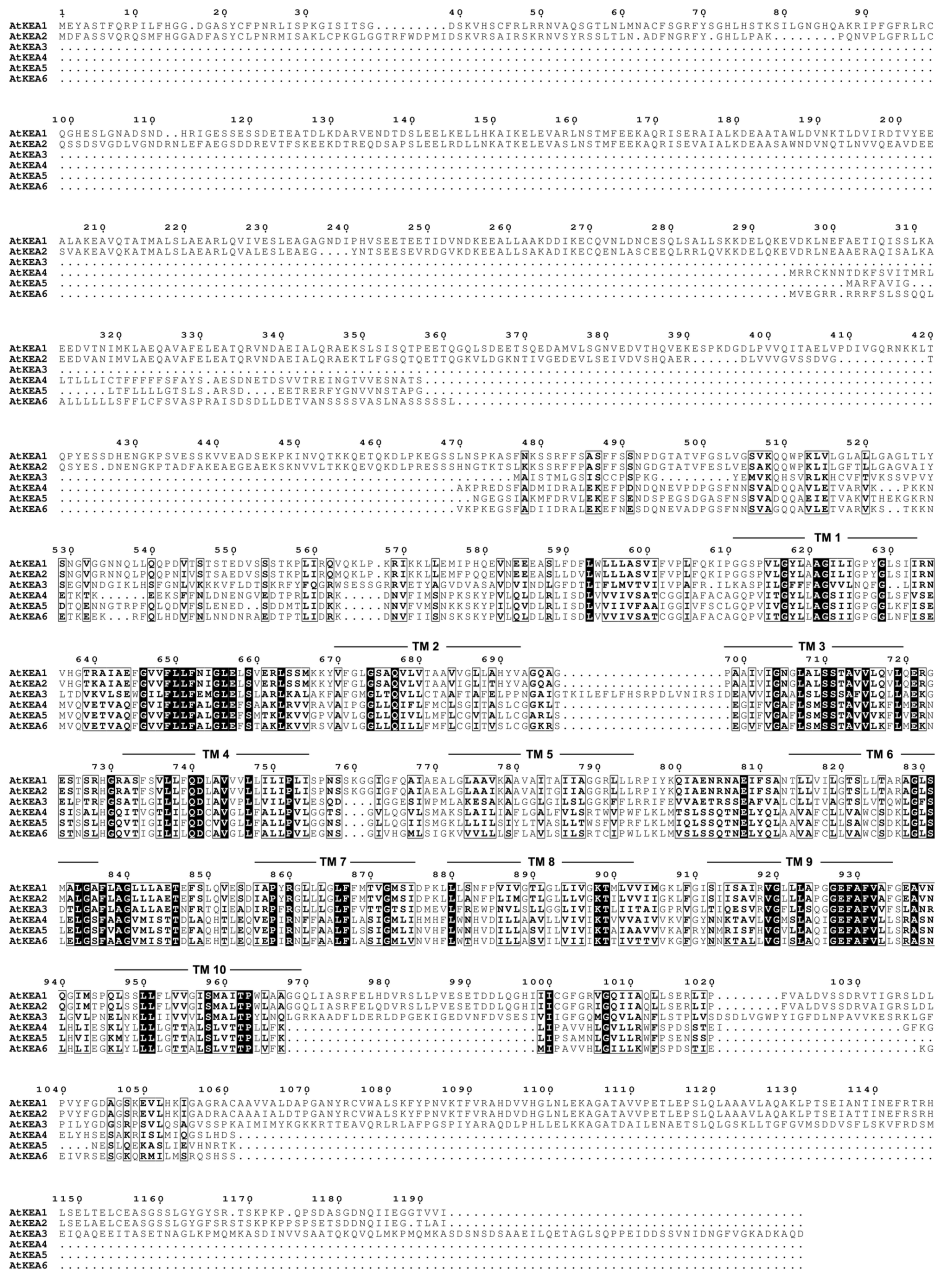
Multiple alignment of the putative amino acid sequences of the AtKEA family. The predicted amino acid sequences of AtKEA1 (AEE27335), AtKEA2 (AEE81911), AtKEA3 (AEE82433), AtKEA4 (AEC06899), AtKEA5 (AED96117) and AtKEA6 (AED91722) were aligned based on analysis using the ClustalX 2.1 method. Identical or similar residues are blocked as dark or light boxes, respectively. Putative transmembrane domains were analyzed using the TMHMM method and are marked at the approximate TM regions.

**Figure 2:**
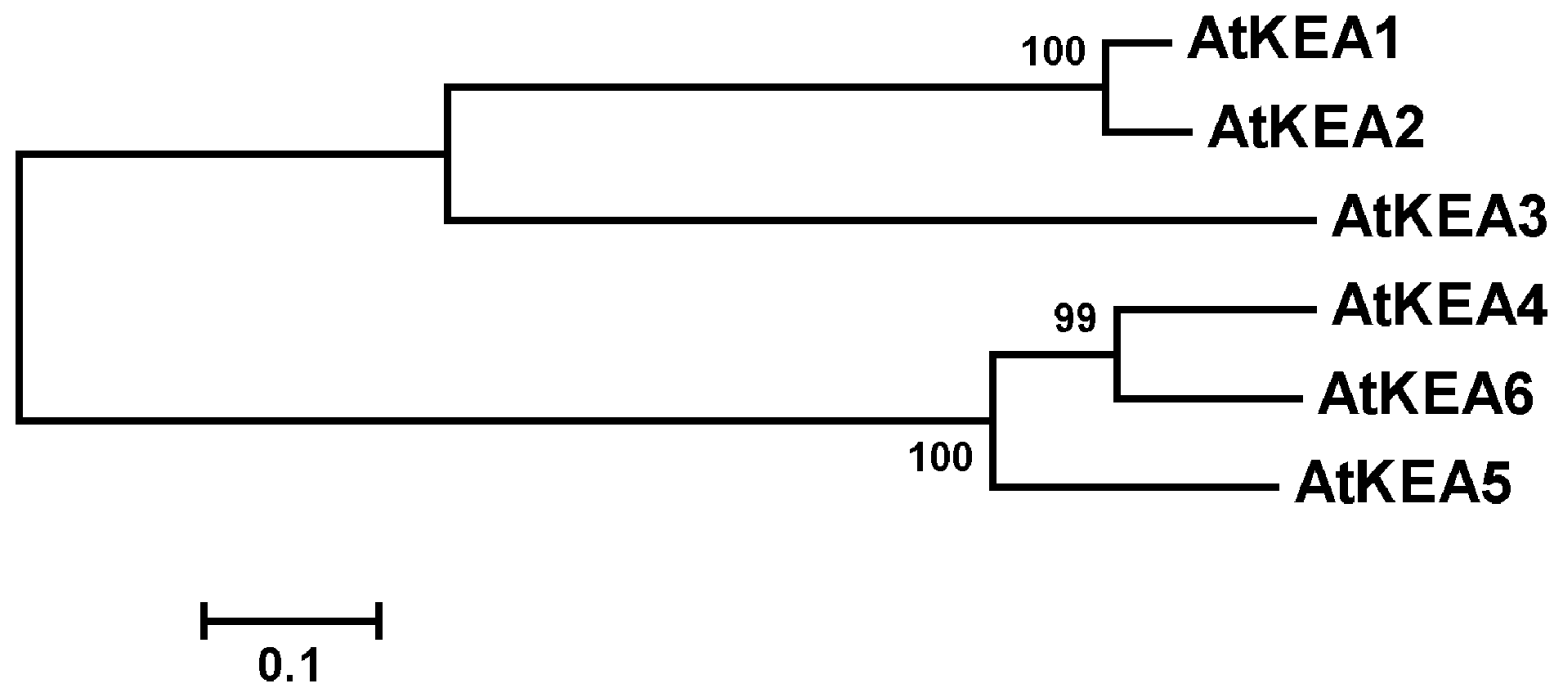
Cladogram analysis of the AtKEA family. Cladogram analysis was conducted using MEGA 5.02. The alignment was based on the predicted amino acid sequences of AtKEA1, AtKEA2, AtKEA3, AtKEA4, AtKEA5 and AtKEA6. Evolutionary distances were computed by the Neighbor Joining method. The scale bar indicates the distance calculated from the multiple alignments.

The Arabidopsis AtKEA gene family contains six members, named *KEA1* through *KEA6* [3], [9]. Based on amino acid sequences, AtKEA are predicted to encode K^+^/H^+^ antiporters with 10 transmembrane spanning domains (Table 1 and Figure 1). The AtKEA genes are named in decreasing order of sequence similarity to AtKEA1, except for AtKEA5 which has a higher similarity (22.4%) than AtKEA4 (21.9%) (Table 1 and Figure 2). AtKEA2-3 are 29.9-84.5% similar to AtKEA1, whereas AtKEA4-6 are 75.0-83.4% similar to each other but only 21.9-30.0% similar to the AtKEA1-3 isoforms (Table 1). The AtKEA gene family forms two subgroups in cladogram, AtKEA1-3 and AtNHX4-6 (Figure 2). 

**Table 1:** Amino acid similarity comparison of the six *Arabidopsis thaliana* family members of AtKEA K^+^/H^+^ antiporters.

	AtKEA1 (AEE27335)	AtKEA2 (AEE81911)	AtKEA3 (AEE82433)	AtKEA4 (AEC06899)	AtKEA5 (AED96117)	AtKEA6 (AED91722)
AtKEA1 (AEE27335)	—	84.5%	29.9%	21.9%	22.4%	24.5%
AtKEA2 (AEE81911)		—	31.0%	23.2%	22.4%	24.9%
AtKEA3 (AEE82433)			—	28.2%	28.6%	30.0%
AtKEA4 (AEC06899)				—	75.2%	83.4%
AtKEA5 (AED96117)					—	75.0%
AtKEA6 (AED91722)						—

The family members are listed in order of sequence similarity beginning with the prototype AtKEA1. Accession numbers corresponding to the GenBank database are given in parentheses.

AtKEA1 and AtKEA2 have a long N-terminal domain, which contains 610 and 590 amino acids, respectively (Figure 1). The N-terminal regions of AtKEA1 and AtKEA2 have been predicted to carry a chloroplast transit peptide [46]. The chloroplast localization of AtKEA1 has been identified by a mass spectrometry assay [47]. The localization of AtKEA2 in chloroplasts has been visualized in seedlings transformed with the GFP tagged AtKEA2 [46]. In addition, AtKEA1 and AtKEA2 have a long C-terminal tail, containing 224 and 222 amino acids, respectively (Figure 1), suggesting that AtKEA1 and AtKEA2 may have a distinct regulatory mechanism. 

**Figure 3:**
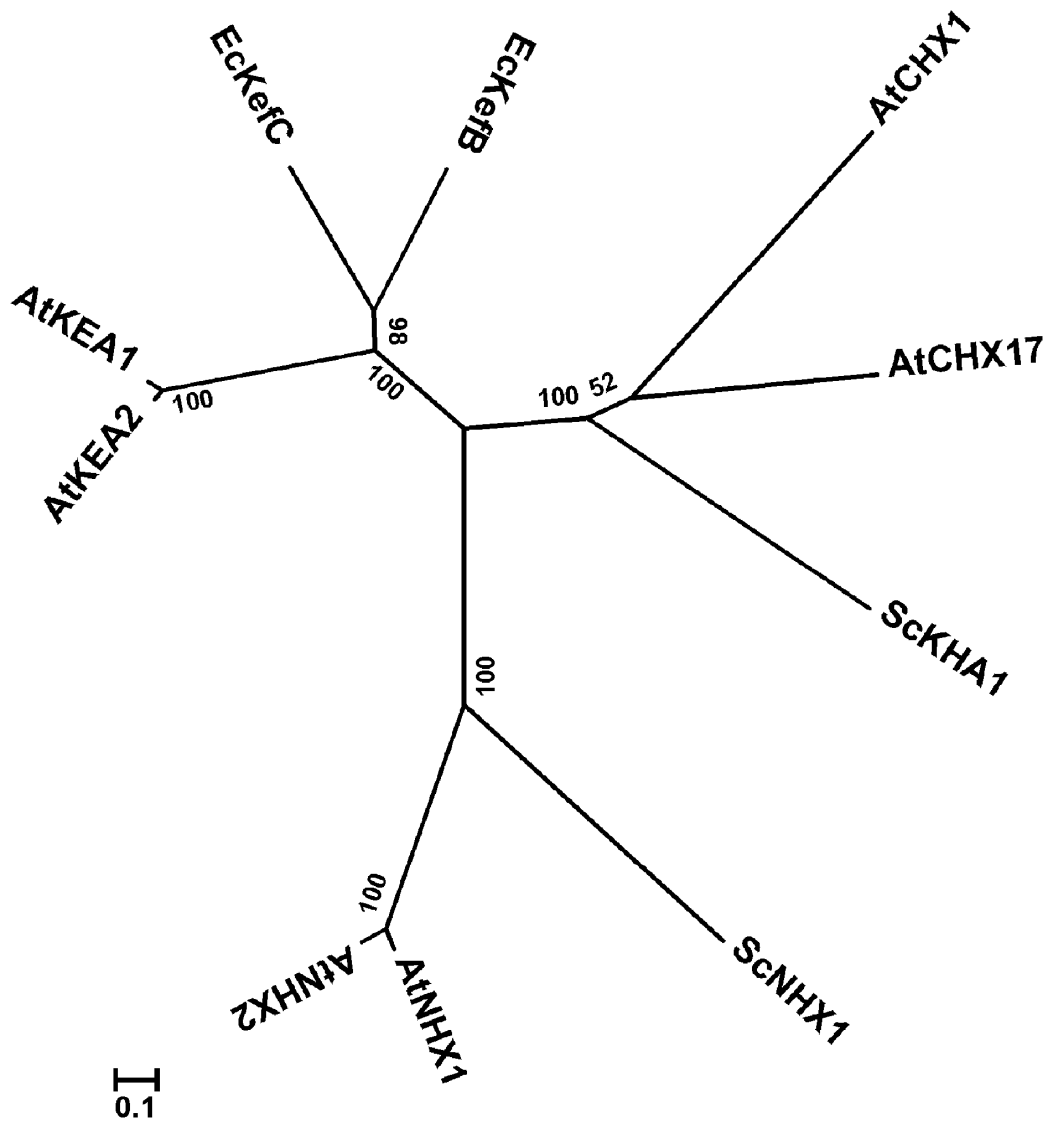
Phylogenetic analysis of AtKEAs, AtNHXs and AtCHXs. Phylogenetic analysis was conducted using MEGA 5.02. The alignment is based on the predicted amino acid sequences. Evolutionary distances were computed by the Neighbor Joining method. The scale bar indicates the distance calculated from the multiple alignment. The accession numbers and sources of the Na^+^,K^+^/H^+^ antiporters are as follows: EcKefB(ACT40850), EcKefC(CAA40066), *Escherichia coli*; ScKHA1(DAA08706), ScNHX1(DAA12290), *Saccharomyces cerevisiae*; AtKEA1, AtKEA2, AtNHX1(AAD16946), AtNHX2(AAM08403), AtCHX1 (AEE29444), AtCHX17 (AEE84796), *Arabidopsis thaliana*.

Phylogenetic analysis shows that AtKEAs form a cluster with their E. coli orthologs EcKefB/EcKefC, separating clearly from the clusters of AtNHXs and AtCHXs with their yeast orthologs (Figure 3). Thus, AtKEAs may function distinctly from either AtNHXs or AtCHXs.

### The full-length AtKEA1 protein is inactive in yeast

Protein organization analysis showed that AtKEA1 and AtKEA2 are comprised of a soluble N-terminal domain, a Na_H exchange domain and a C-terminal KTN domain [4], [46]. The long N-terminal domains of AtKEA1 and AtKEA2 were missed in early gene annotation. The cDNA sequences of the short version AtKEA1 and AtKEA2, AtsKEA1 and AtsKEA2 , lacking the N-terminal domains but containing the Na_H exchange domains, have been cloned in yeast by Dr. John Ward lab. 

We attempted to clone the full-length cDNAs of AtKEA1 and AtKEA2 genes. Since the direct amplification from the Arabidopsis cDNA preparation was not successful, we used a two-step strategy. We separated the gene into two pieces by choosing a restriction enzyme site in the middle of the gene; the two pieces were cloned separately. We then combined them to get the full-length cDNA. For AtKEA1, an EcoRI site was chosen to separate the gene into two pieces of A_1_-C_1870_ and T_1871_-A_3582_, and we successfully cloned the full-length cDNA. However, using the same strategy, we did not clone the full length AtKEA2 gene. Aranda-Sicilia et al. (2012) also failed to clone the full-length AtKEA2 in their study.

The activity of the full-length AtKEA1 was tested in yeast. The full-length AtKEA1 did not confer resistance to high K^+^ and hygromicin B (50 ug/ml) in yeast growth compared with AtsKEA1 (Figure S1). Moreover, the full-length AtKEA1 did not confer resistance to high K^+^ at pH 4.5 relative to AtsKEA1 (Figure S2). Thus, the full-length AtKEA1 is inactive in yeast, whereas the AtsKEA1 is functional, suggesting that the N-terminal end of AtKEA1 may be an autoinhibitory domain controlling the transport activity.

The subcellular localization of the full-length AtKEA1 was detected by expressing AtKEA1-GFP in yeast cells (Figure S3). The GFP fluorescence did not appear at clear cellular structures; instead, the proteins formed structureless clumps in yeast cells, suggesting that the full-length AtKEA1 did not distribute properly in yeast cells. 

### AtKEAs specifically mediate K^+^ transport in yeast

**Figure 4:**
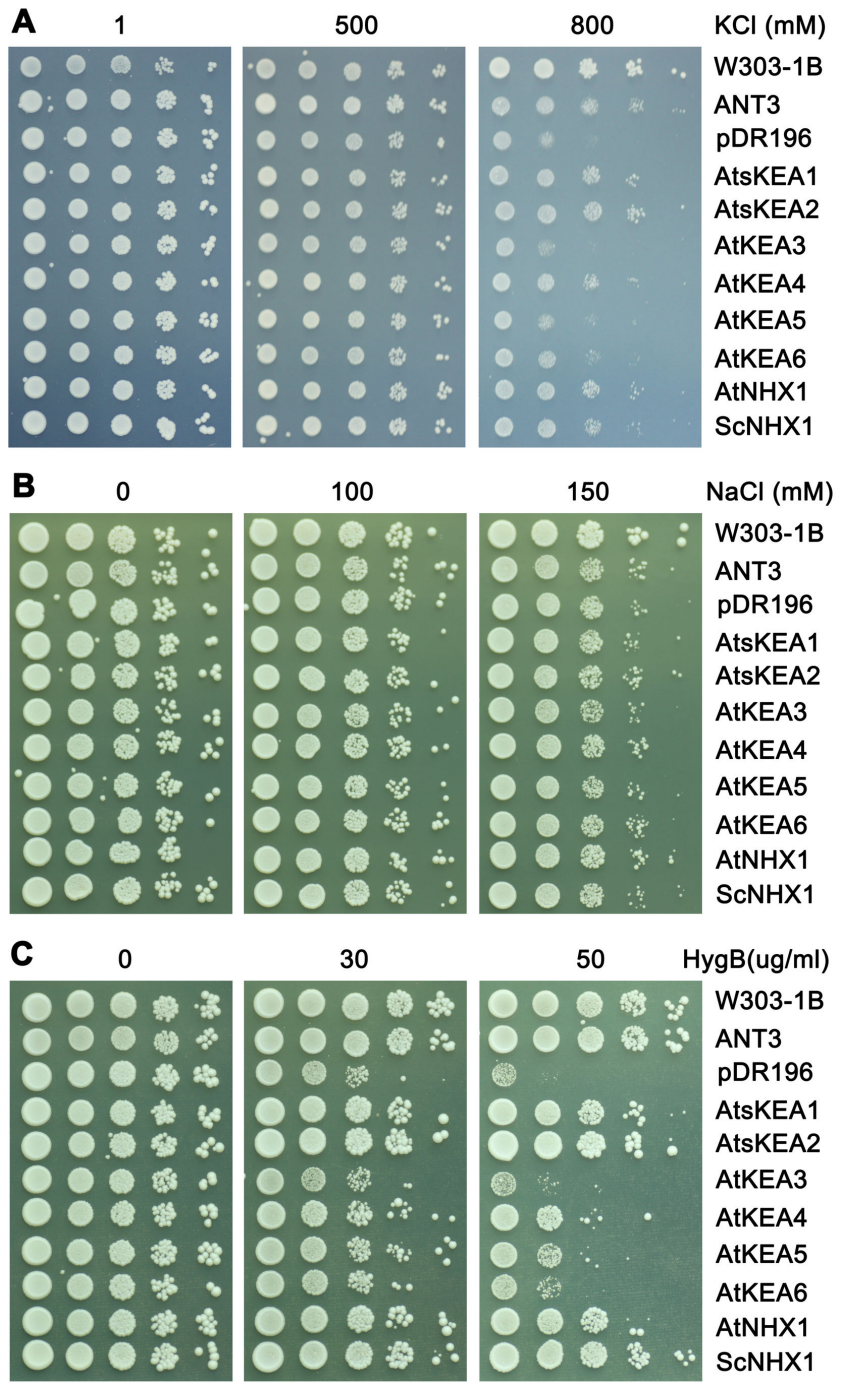
AtKEAs mediate K^+^ transport and confer resistance to hygromycin B in yeast mutants. The cDNAs of AtKEAs, AtNHX1 and ScNHX1 were subcloned into the yeast expression vector pDR196 and transformed into the AXT3 mutant (ena1-4 nha1 nhx1). Yeast cells were normalized in water to A_600_ of 0.4. Aliquots (4μL) from normalized yeast cultures or 10-fold serial dilutions were spotted onto AP plates containing different concentrations of KCl (A), or YPD plates with different concentrations of NaCl (B), or hygromycin B (C). The strains were grown at 30°C for 3 days.

To test the function of AtKEAs, the coding sequences of *AtKEA1-6* were cloned in the yeast expression vector pDR196 and introduced into a *Saccharomyces cerevisiae* strain AXT3. Strain AXT3 lacks the functional plasma membrane Na^+^-ATPases (ScENA1-4), plasma membrane Na^+^,K^+^/H^+^ antiporter ScNHA1, and vacuolar Na^+^,K^+^/H^+^ antiporter ScNHX1. Thus, it is sensitive to salt and to high K^+^. The transformed yeast was grown on Arg phosphate (AP) medium with high levels of KCl or NaCl (Figure 4). AXT3 mutants failed to grow in the medium containing 800 mM KCl while the *nhx1*-positive strains (W303-1B and ANT3) grew vigorously (Figure 4A). Expression of AtsKEA1 and AtsKEA2, AtKEA4, -5 and -6 recovered tolerance to high K^+^, similar to the AXT3 strains expressing ScNHX1 or AtNHX1 (Figure 4A). However, the recovery capacities among the AtKEA family were different. AtsKEA1 and AtsKEA2 had the highest effect, whereas AtKEA3 had no effect (Figure 4A). Interestingly, although AtKEA genes were well expressed in AXT3 mutants as detected with GFP tagged proteins (Figure S5), yeast growth was not improved in salt stress (Figure 4B), suggesting that AtKEAs do not confer tolerance to salt stress. In addition, AtKEAs did not confer Li^+^ tolerance (data not shown). AXT3 mutants were shown to be sensitive to hygromicin B (50 μg/ml), and ScNHX1 and AtNHX1 enhanced tolerance to hygromicin B (Figure 4C). While all AtKEA genes conferred resistance to the drug hygromicin B, AtsKEA1 and AtsKEA2 had the most effect (Figure 4C), suggesting their roles in endosomal compartments. These results suggest that AtKEAs specifically facilitate K^+^ homeostasis, which is dissimilar to AtNHXs. However, AtKEAs are similar to both AtNHXs and AtCHXs in that all three function in endosomal trafficking.

### AtKEAs have strict pH requirements in mediating K^+^ transport in yeast

**Figure 5:**
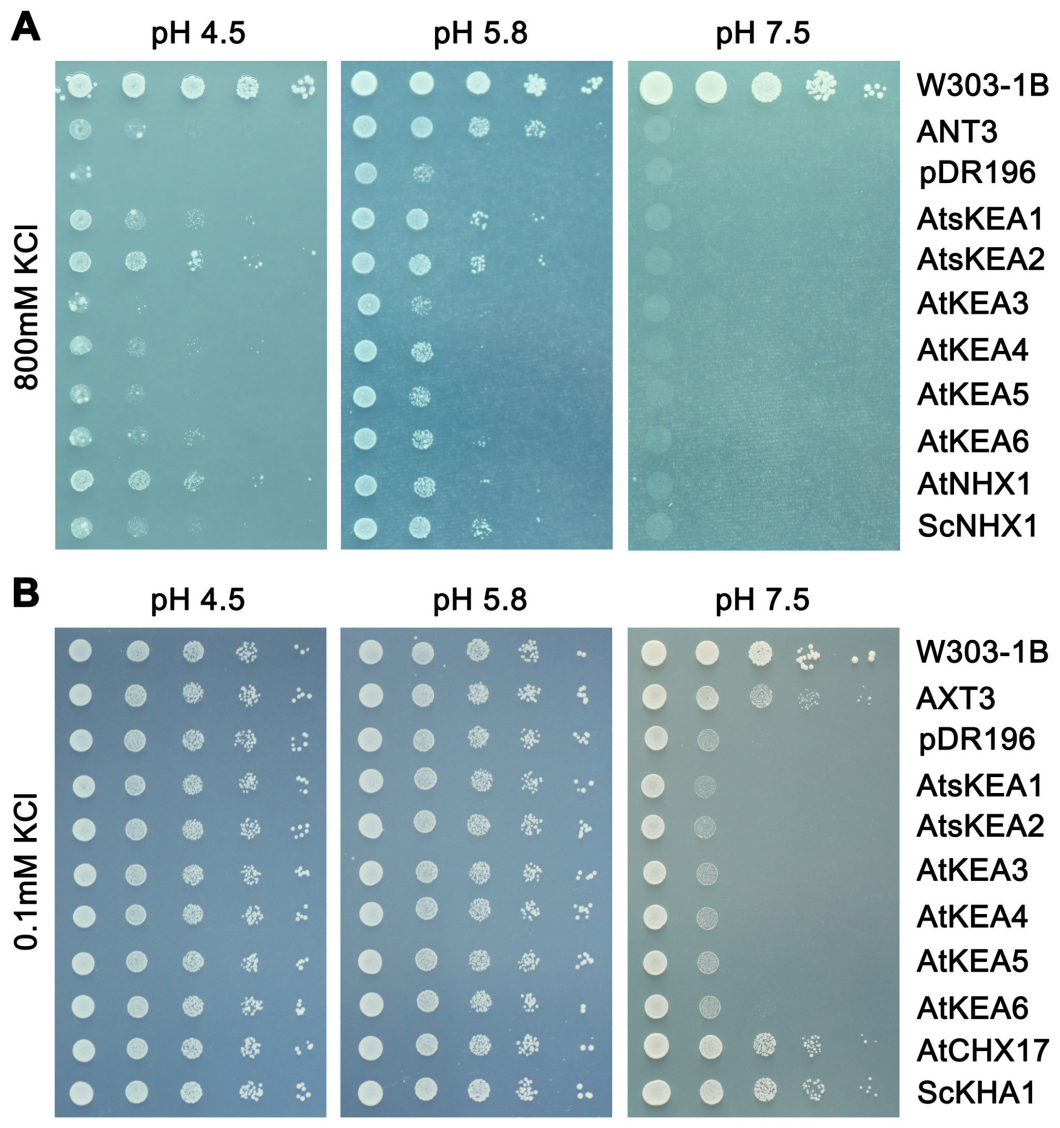
AtKEAs have strict pH requirements in mediating K^+^ transport in yeast. (A) The cDNAs of AtKEAs, AtNHX1and ScNHX1 were subcloned into the yeast expression vector pDR196 and transformed into strain AXT3 (ena1-4 nha1 nhx1). Strains were spotted onto AP plates containing 800mM KCl at pH 4.5, 5.8 or 7.5. (B) The cDNAs of AtKEAs, AtCHX17 and ScKHA1 were subcloned into the yeast expression vector pDR196 and transformed into strain AXT4K (ena1-4 nha1 nhx1 kha1). Strains were spotted onto AP plates containing 0.1mM KCl at pH 4.5, 5.8 or 7.5. Cells were normalized in water to A_600_ of 0.4. Aliquots (4μL) from normalized yeast cultures or 10-fold serial dilutions were spotted onto AP plates. The strains were grown at 30°C for 3 days.

AtKEA genes (except AtKEA3) recovered AXT3 mutant growth at 800 mM KCl at an external pH of 5.8 (Figure 5A). However, the recovery capacity of the AtKEA family was significantly reduced when pH was dropped to 4.5 under 800 mM KCl, while AtNHX1 and ScNHX1 were still active under the same conditions. Furthermore, AtKEAs completely lost their functions at pH 7.5 at 800 mM KCl (Figure 5A). Thus, AtKEAs require a specific pH in mediating K^+^ transport in yeast; either alkaline or more acidic conditions will affect their functions. AtKEAs were further tested in the yeast mutant strain AXT4K, generated by deleting *kha1* in the AXT3 mutant background. AXT4 mutants failed to grow at low K^+^ at pH 7.5, while the *kha1*-positive strains (W303-1B and AXT3) grew dynamically (Figure 5B). However, expression of AtKEAs failed to rescue AXT4K growth at low K^+^ at pH 7.5, while ScKHA1 and AtCHX17 enhanced yeast growth (Figure 5B). These results indicate that AtKEAs, AtNHXs and AtCHXs may have different modes of action in facilitating K^+^ homeostasis. AtKEAs function at high K^+^ at pH 5.8 while AtNHXs function at high K^+^ in acidic environments and AtCHXs at low K^+^ under alkaline conditions. 

### *AtKEAs* express in both the shoot and root of Arabidopsis

**Figure 6:**
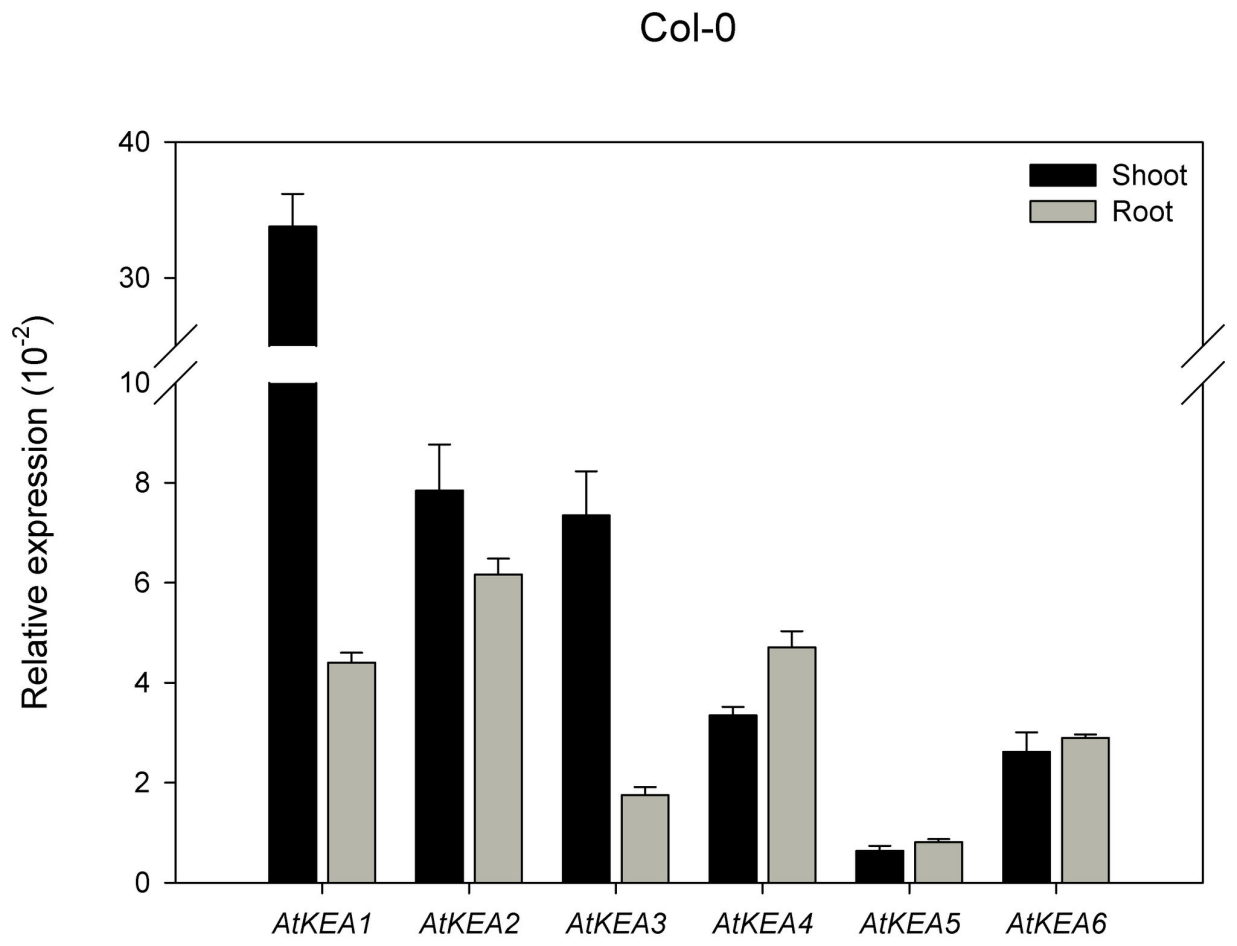
The expression of AtKEAs in shoots and roots of Arabidopsis seedlings. Fourteen-day-old *Arabidopsis* seedlings (Col-0) were used. The relative transcript abundance of AtKEAs in shoots and roots were analyzed by RT-qPCR. Actin7 gene was used as an internal positive control. Error bars represent SE (n =3).

The transcript abundances of *AtKEA* genes were analyzed by RT-qPCR using gene-specific primers. Shoots and roots were isolated from 14-day-old WT seedlings growing on 1/2 MS medium. The transcripts of *AtKEA* genes were detected in shoots and roots (Figure 6). *AtKEA2, -4, -5* and *-6* had almost equal expression in shoots and roots; *AtKEA1* and *AtKEA3*, however, showed a relatively higher expression in shoots than in roots, suggesting their dominant roles in shoots. *AtKEA5* had the lowest expression in both shoots and roots. The differential expression indicates that *AtKEA* genes have diversified roles in Arabidopsis.

### *AtKEAs* respond differently to low K^+^ stress

**Figure 7:**
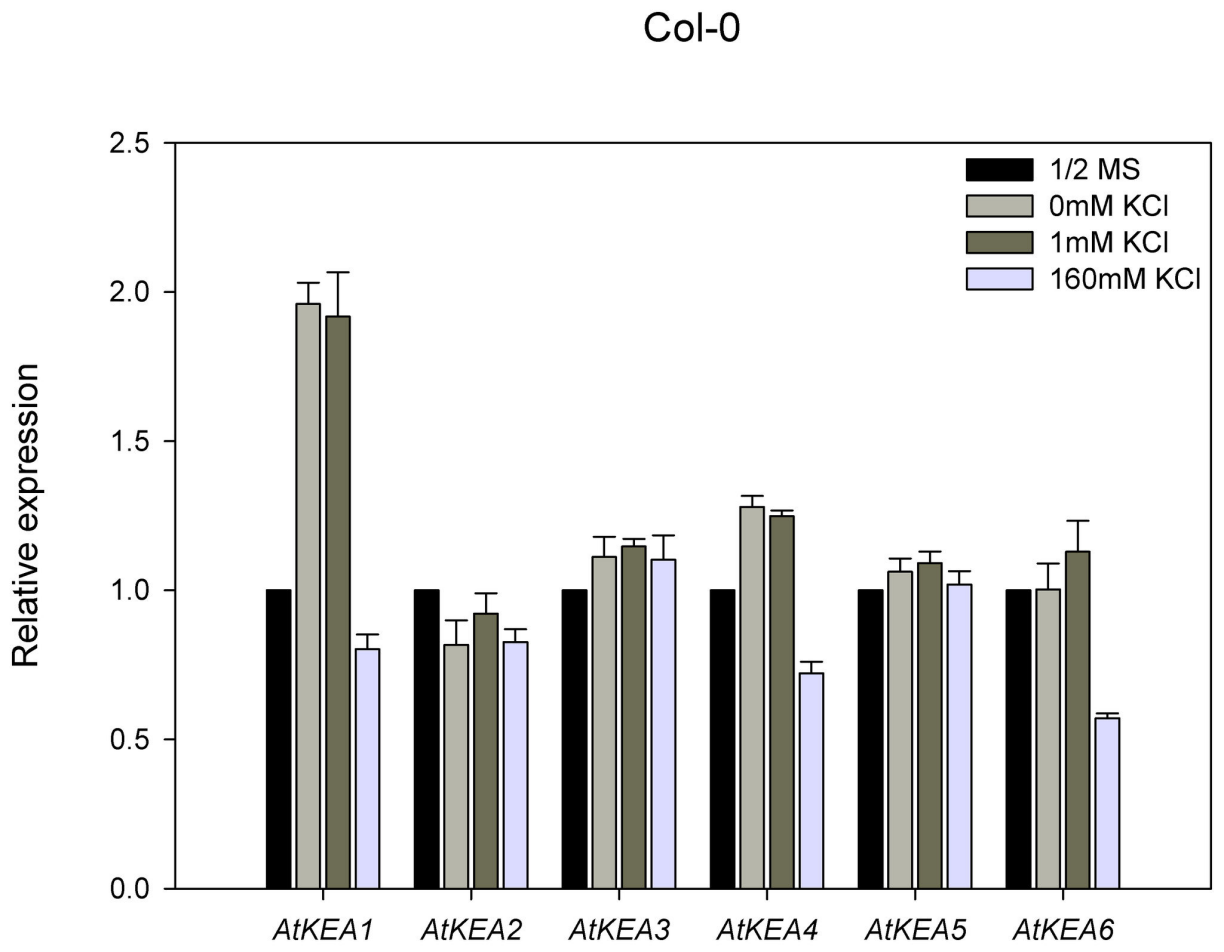
The expression of AtKEAs responds differently to low K^+^ treatment. Fourteen-day -old *Arabidopsis* seedlings (Col-0) were cultured on the 1/2 MS medium or modified MS without K^+^ (0 mM K^+^) or supplemented with 1mM or 160mM KCl for 8h. Actin7 gene was used as an internal positive control. The transcript levels of AtKEAs in the untreated control seedlings were set as 1.0. Error bars represent SE (n=3).

Since yeast growth assays have shown that AtKEAs mediate K^+^ transport (Figure 4A and 5A), we tested whether *AtKEA* expression was induced by K^+^. Interestingly, *AtKEA1* expression was enhanced significantly under low K^+^ stress (0 and 1 mM K^+^) compared to the untreated control (Figure 7). The expression of AtKEA3 and AtKEA4 was also enhanced under low K^+^ stress, but AtKEA2, -5, and -6 was not. In contrast, gene expressions were significantly reduced for AtKEA1, -2, -4 and -6 at high K^+^ (160 mM) (Figure 7). The differential expression in response to low K^+^ stress suggests that AtKEA1, -3,and -4 are involved in K^+^ acquisition under low K^+^ conditions in Arabidopsis, whereas AtKEA2, -5 and -6 may have different functions. 

### The expression of *AtKEA2* and *AtKEA5* is induced by osmotic stress and is dependent on ABA signaling

**Figure 8:**
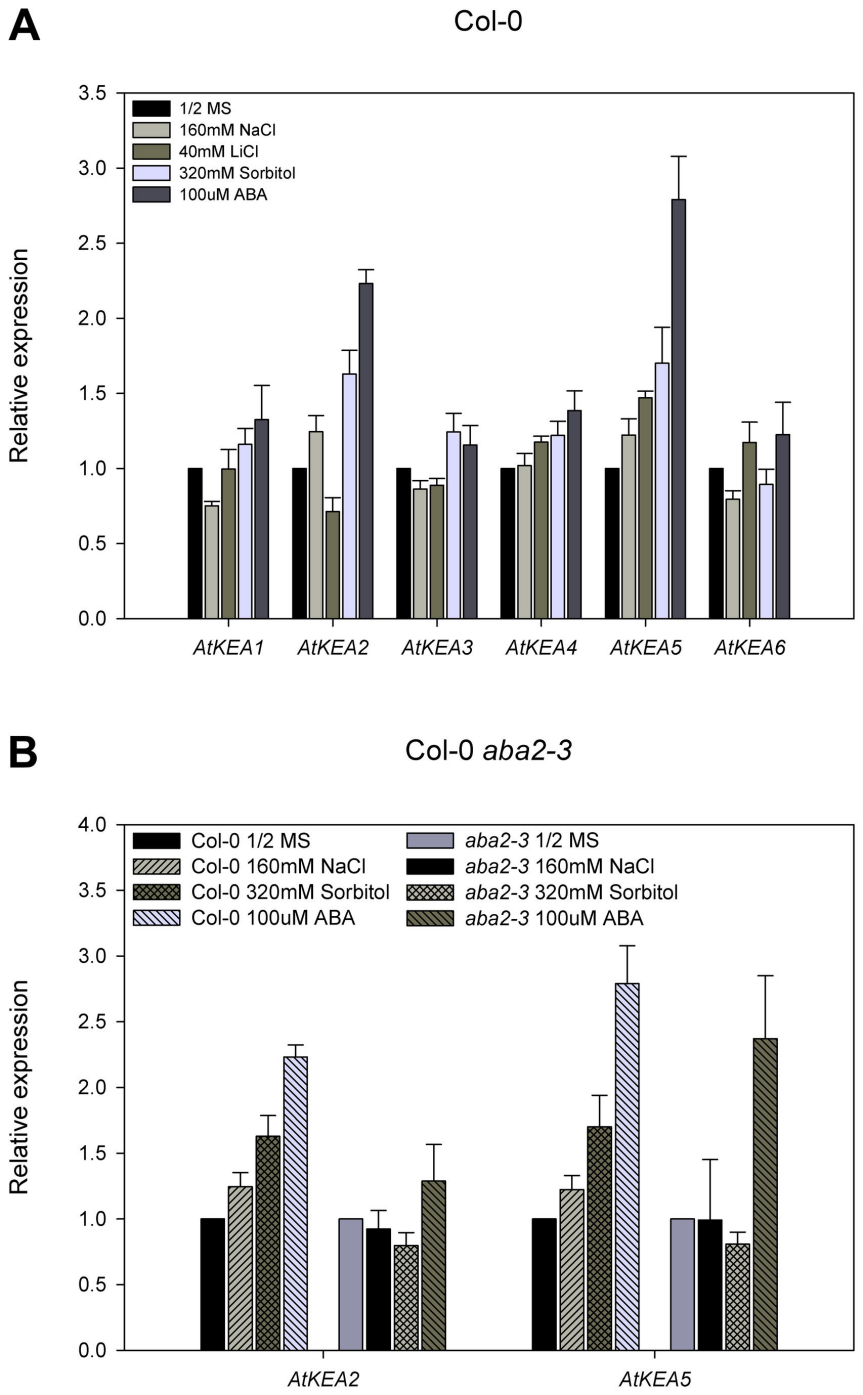
The expression of AtKEA2 and AtKEA5 is strongly enhanced under osmotic and ABA treatments. *Arabidopsis* seedlings (Col-0, *aba2-3*) were grown on 1/2MS-agar media for 14 days. (A) Col-0 were continued culturing on the fresh 1/2 MS medium without or supplemented with 160 mM NaCl, 40 mM LiCl, 320 mM Sortibol, or 100 μM ABA, respectively, for 8h. (B) Col-0 and *aba2-3* were continued culturing on the fresh 1/2 MS medium without or supplemented with 160 mM NaCl, 320 mM Sortibol or 100μM ABA, respectively, for 8h. Actin7 gene was used as an internal positive control. The transcript levels of AtKEAs in the untreated control seedlings were set as 1.0. Error bars represent SE (n =3).

*AtKEA* expression was further tested under Na^+^, Li^+^ and sorbitol stresses. Interestingly, the expression of *AtKEA2* and *AtKEA5* was strongly induced under 320 mM sorbitol and 100 μM ABA treatments compared to their untreated controls, indicating that these two genes were osmotic responsive and were ABA-dependent (Figure 8A). *AtKEA2* and *AtKEA5* were also induced by 160 mM NaCl, which was iso-osmotic to 320 mM sorbitol (Figure 8A), suggesting that *AtKEA2* and *AtKEA5* are responsive to osmotic stress other than ionic stress. In addition, the expression of *AtKEA4* and *AtKEA5* were induced by Li^+^ stress compared to their untreated controls, while no induction was observed for *AtKEA1, -2, -3* and *-6* (Figure 8A). 

ABA regulation of *AtKEA2* and *AtKEA5* expression was further tested with *aba2-3* mutant. The *aba2-3* mutant is deficient in ABA synthesis but is responsive to exogenously applied ABA [61]. Under iso-osmotic NaCl and sorbitol treatments, while the expression of *AtKEA2* and *AtKEA5* was induced in WT plants, it was not induced in *aba2-3* mutants compared to the untreated control (Figure 8B). However, ABA treatments strongly induced the expression of both *AtKEA2* and *AtKEA5* in *aba2-3* mutants relative to their untreated controls (Figure 8B). These results suggest that the induction of *AtKEA2* and *AtKEA5* by osmotic stress is dependent on ABA signaling.

### *AtKEAs* are not regulated by the SOS pathway

**Figure 9:**
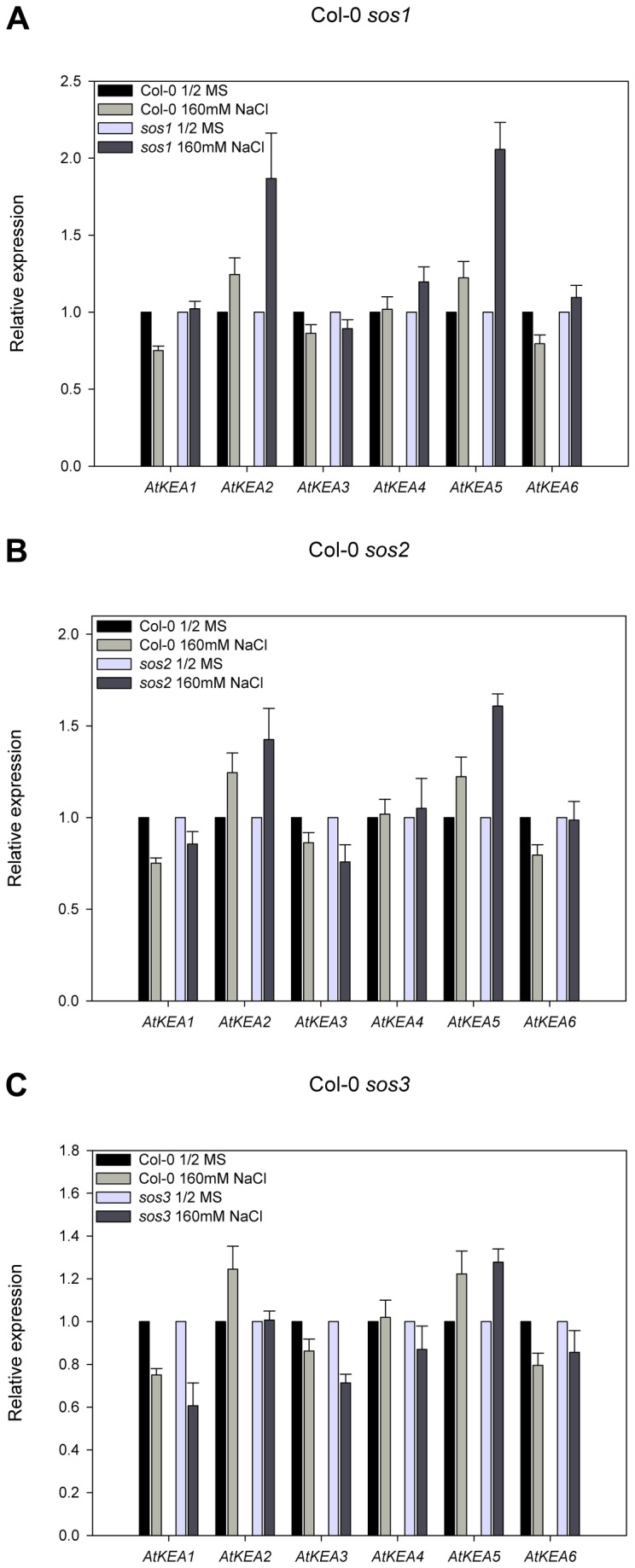
The expression of AtKEAs is not controlled by the SOS signaling pathway. *Arabidopsis* seedlings (Col-0, *sos1-1*, *sos2-1* or *sos3-1*) were grown on 1/2MS-agar media for fourteen days. Seedlings were continued culturing on the fresh 1/2 MS medium without or supplemented with 160 mM NaCl, respectively, for 8h. (A) Col-0 and *sos1-1*; (B) Col-0 and *sos2-1* ; (C) Col-0 and *sos3-1*. Actin7 gene was used as an internal positive control. The transcript levels of AtKEAs in the untreated control seedlings were set as 1.0. Error bars represent SE (n =3).

The Arabidopsis thaliana Salt Overly Sensitive (SOS) pathway controls ion homeostasis in plants [62], [63]. The SOS pathway is composed of SNF-like kinase SOS2 and Ca^2+^-binding protein SOS3. SOS3 perceives the ion stress signals and activates SOS2. SOS2 in turn activates SOS1, a plasma membrane Na^+^/H^+^ antiporter in Arabidopsis [15], [17], [62]. In an attempt to understand whether *AtKEA* expression is regulated by the SOS pathway, we tested *AtKEA* expression in *sos* mutants by RT-qPCR (Figure 9). Interestingly, the expression of the *AtKEA* family in *sos1*, *sos2*, or *sos3* mutants was not affected by salt stress compared to their WT plants with salt stress, suggesting that *AtKEAs* were not controlled by the SOS pathway under salt stress. On the contrary, *AtKEA5* expression in *sos1*and *sos2* mutants was consistently high compared to either the WT plants with salt stress or their untreated controls. Similarly, *AtKEA2* expression in *sos1*and *sos2* mutants was high relative to their untreated controls (Figure 9A, 9B). These results suggest that *AtKEA2* and *AtKEA5* may be regulated negatively by the SOS pathway. Or, the induction of *AtKEA2* and *AtKEA5* is caused by the osmotic stress generated by the accumulation of salt in *sos* mutants. 

### AtKEAs distribute diversely in yeast cells

**Figure 10:**
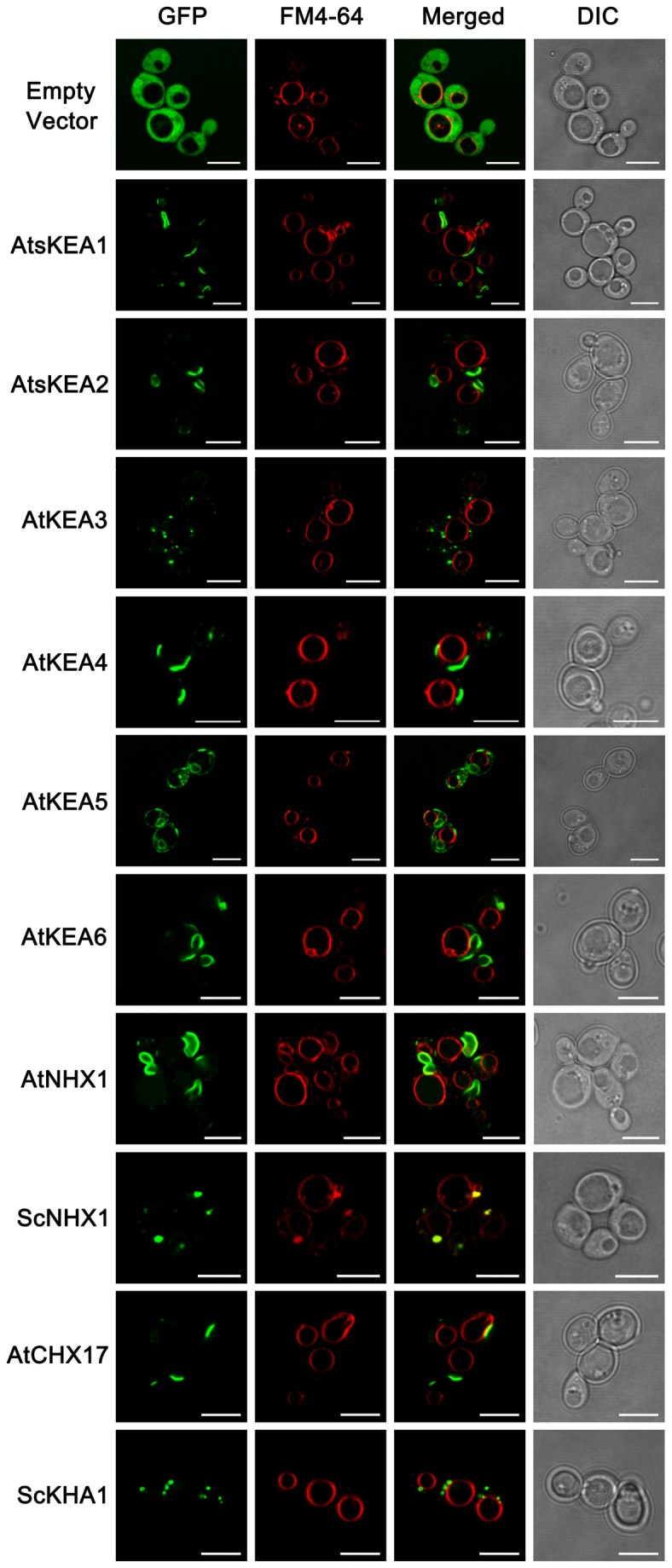
The diversified distribution of AtKEAs in yeast. Wild-type (W303-1B) yeast strains harboring pDR196-GFP, pDR196-AtKEAs-GFP, pDR196-AtNHX1-GFP, pDR196-AtCHX17-GFP, pDR196-ScNHX1-GFP, and pDR196-ScKHA1-GFP (fused with GFP at the C terminus), respectively, were grown to logarithmic phase in SC-URA medium (pH 5.8) and were stained with FM4-64 dye. The subcellular localizations of the GFP-tagged proteins (green) and FM4-64 fluorescence (red) were observed under the Laser Scanning Confocal Microscope. Bars, 5µm.

AtKEAs fused with GFP at the C-terminals retained resistance to hygromicin B in yeast (Figure S4). In addition, the GFP-tagged AtKEAs distributed properly in the transport activity test strains AXT3 and shared the same pattern as that in wild-type yeast (W303-1B) (Figure S5, Figure 10). These results demonstrate that the AtKEAs tagged with GFP at their C-terminals retained activity in yeast cells.

In wild-type yeast (W303-1B), AtsKEA1-GFP, AtsKEA2-GFP and AtKEA6-GFP had similar distribution patterns; the fluorescent signals appeared at a structure at or near the plasma membrane and a membrane structure in the cytosol. These structures were not overlapped with FM4-64 (Figure 10). The distribution patterns of AtsKEA1-GFP, AtsKEA2-GFP and AtKEA6-GFP were similar to AtNHX1 (Figure 10). However, AtKEA3-GFP shared similar patterns with ScKHA1; the fluorescent signals appeared at punctate structures (Figure 10). ScKHA1 has been determined to be localized to Golgi [64], [65], indicating that AtKEA3-GFP is localized to Golgi in the yeast cells. AtKEA4-GFP shared similar patterns with AtCHX17; the fluorescent signals appeared at a membrane structure in the cytosol that was not overlapped with FM4-64 (Figure 10). AtKEA5-GFP signals appeared at both punctate structures and a membrane structure in the cytosol; neither structure was overlapped with FM4-64 (Figure 10). These patterns suggest that AtKEAs have different distributions in yeast cells. 

### AtKEA3 is localized to Golgi in Arabidopsis

**Figure 11:**
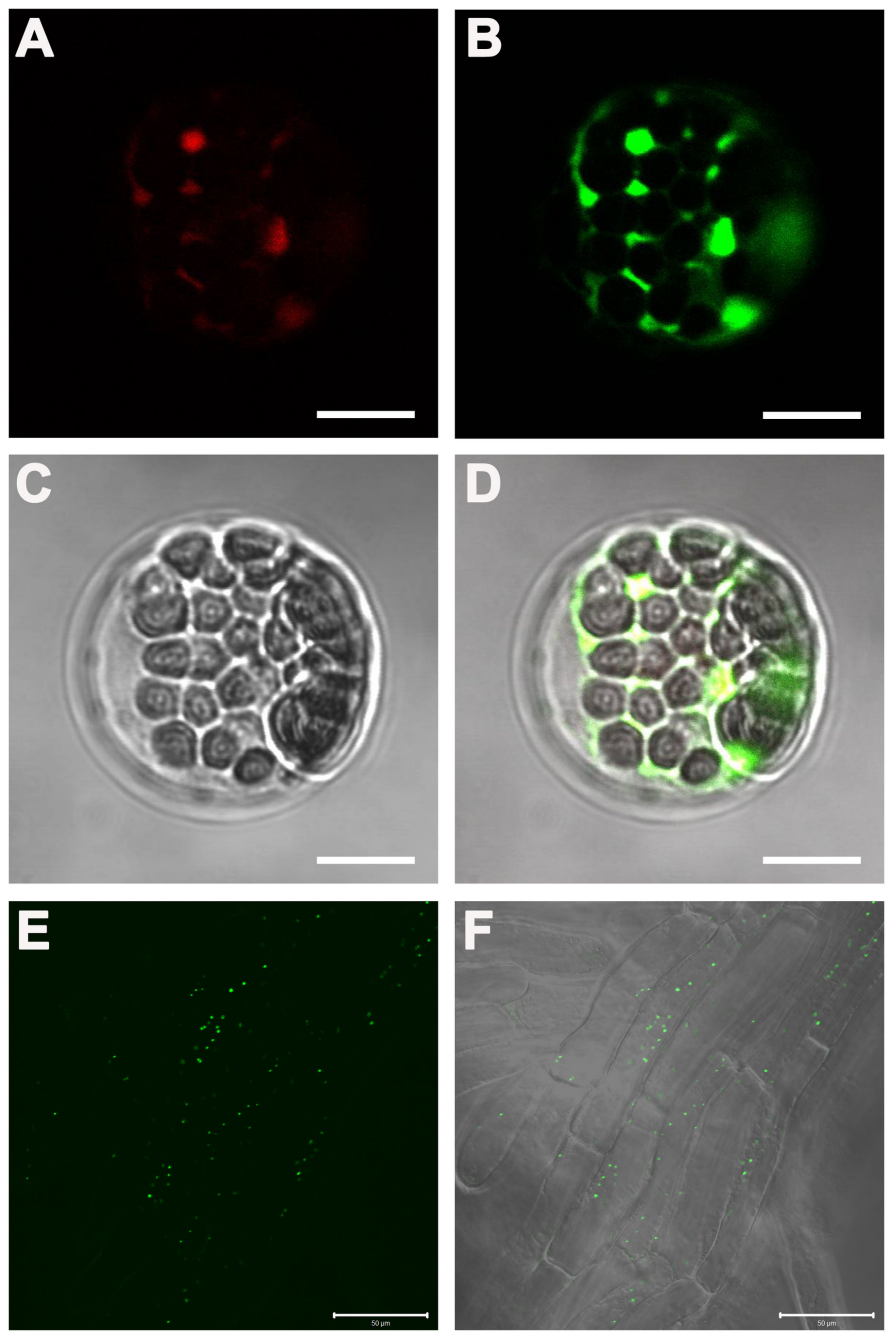
AtKEA3 is localized to Golgi in Arabidopsis. (A), (B), (C) and (D): The transient expression of AtKEA3 in Arabidopsis protoplasts. A cis-Golgi marker GFP-AtSYP31 and RFP-AtKEA3 were co-transformed in Arabidopsis (Col-0) protoplasts. (A): RFP-AtKEA3 fluorescence image; (B): GFP-AtSYP31 fluorescence image; (C): Transmission image; (D): Overlay of the fluorescence and transmission images. Bars, 10µm. (E) and (F): The localization of AtKEA3 in root cells of the stably transformed Arabidopsis seedlings. (E): GFP fluorescence image; (F): Overlay of the fluorescence and transmission images. Images were observed under the Laser Scanning Confocal Microscope. Bars, 50µm.

We visualized the subcellular localization of AtKEA3 by transient expression in Arabidopsis protoplasts. Surprisingly, RFP-AtKEA3 fluorescence appeared on punctate structures in the cytosol but not in chloroplasts (Figure 11A). RFP-AtKEA3 fluorescent signals were co-localized extensively with the Golgi marker GFP-AtSYP31, suggesting that AtKEA3 is localized to Golgi (Figure 11B, D). 

To verify the subcellular localization of AtKEA3, we generated stably transformed Arabidopsis seedlings expressing cauliflower mosaic virus 35S-driven AtKEA3-GFP. In consistent with the transient expression assay, AtKEA3-GFP fluorescent signals were visualized at the punctate structures within the cells (Figure 11E, F). These punctate structures are the typical structures of Golgi bodies [66], [67], indicating that AtKEA3 is localized to the Golgi membranes. However, no fluorescent signals appeared in chloroplasts (Figure 11E, F). These studies from both yeast and plants suggest that AtKEA3 is localized to Golgi. 

## Discussion

### AtKEAs may facilitate K^+^ homeostasis and play diversified roles in Arabidopsis

In this report, we presented the first experimental characterization of the novel AtKEA gene family, putative K^+^/H^+^ antiporters in Arabidopsis. We showed that AtKEAs (except AtKEA3) conferred resistance to high K^+^ stress in yeast mutants (Figure 4, 5), and the expression of *AtKEA1, -3*, and *-4* were induced under low K^+^ stress (Figure 7). A previous study also showed that AtKEA2 had K^+^ transport activity in a reconstituted liposome assay [46]. Therefore, similar to their bacterial homologs EcKefB/EcKefC, AtKEAs may encode K^+^/H^+^ transporters that function in facilitate K^+^ homeostasis in plants. 

Studies have shown that members of the CPA2 family have various catalytic modes [10]. For example, although EcKefB and EcKefC were thought to function as K^+^/H^+^ antiporters, they share structural similarities with K^+^ channels and act like ligand-gated K^+^ efflux channels [68], [69]. In addition, while the AtCHX family was predicted to encode cation/H^+^ exchangers, studies have shown that AtCHX20 might be a K^+^/H^+^ symporter and AtCHX17 might function as a K^+^ channel [33]. Currently, the catalytic mechanisms for the ion transport activities of AtKEAs remain unclear. The observations that AtKEAs have diversified capacities in conferring resistance to ion, hygromycin B, and pH stresses in yeast (as discussed below) suggest that AtKEAs may have diversified action modes.

Our results suggest that the members of the AtKEA family may function diversely in plants (1). AtKEAs had various capacities in conferring resistance to high K^+^ and hygromycin B in yeast growth (Figure 4); (2) AtKEAs diversely enhanced yeast growth at acidic condition (pH 4.5) (Figure 5A); (3) *AtKEAs* displayed a diversified patterns of expression in shoots and roots, and were induced differently under low K^+^ or osmotic stress (Figures 6, 7, 8). (4) AtKEAs localized diversely in yeast cells (Figure 10). These diversified patterns of K^+^ transport, expression and localization may imply that AtKEAs play diversified roles in different cellular processes under different growth and developmental or environmental conditions. 

### AtKEA2 and AtKEA5 function in osmotic adjustment regulated by the ABA signaling pathway

We showed that the expression of *AtKEA2* and *AtKEA5* were strongly induced by sorbitol treatment (Figure 8A), implying that AtKEA2 and AtKEA5 may function in osmotic adjustment in plants. The response of *AtKEA2* and *AtKEA5* to osmotic stresses may be regulated by the ABA signaling pathway, since we found that the expression of *AtKEA2* and *AtKEA5* was induced by ABA treatment (Figure 8A). This is supported by the assay with the ABA deficient mutant *aba2-3* in which *AtKEA2* and *AtKEA5* were not induced by osmotic stresses (Figure 8B), indicating requirement of the ABA signal for the osmotic response. Indeed, *AtKEA2* and *AtKEA5* contain ABA responsive elements (ABRE) TACGGTC and TACGTGTC, respectively, in their promoter regions (1.5 kb upstream of the translation start codon). However, *AtKEA2* and *AtKEA5* were not controlled by the SOS pathway since gene expression was not altered in *sos* mutants (Figure 9). An earlier study showed that *AtNHX1* and *AtNHX2* were regulated by the ABA signaling pathway in response to osmotic stress. Similarly, *AtNHX1* and *AtNHX2* were not regulated by the SOS pathway [70]. Therefore, *AtKEA2* and *AtKEA5* may share a similar mechanism to *AtNHX1* and *AtNHX2* in response to osmotic stress. 

AtKEA2 has been visualized to be localized in chloroplasts, implying its critical roles in chloroplasts [46]. The chloroplast is a K^+^ pool in cells which contain around 100-200 mM K^+^. K^+^ is important for the electrical balance across the thylakoid membranes and H^+^ homeostasis in the stroma [71], [72]. In addition, as the major inorganic osmolyte in plant cells, K^+^ maintains the structural and volume integrity of chloroplasts in response to light, osmotic stress or water deficit stresses [73]. Thus, our findings regarding its role in K^+^ transport and osmotic adjustment suggest that AtKEA2 may function in facilitating electrical balance and pH hometasis in chloroplasts and maintaining chloroplast structural integrity under osmotic stress. 

### AtKEA3 functions in Golgi

Our results suggest that AtKEA3 is localized to Golgi. First, in yeast cells, the GFP-tagged AtKEA3 appeared at punctuate structures that are similar to the yeast ScKHA1, a Golgi localized K^+^/H^+^ antiporter (Figure 10). Second, in transiently expressed Arabidopsis protoplasts, the RFP-tagged AtKEA3 was well merged with the Golgi marker AtSYP31 (Figure 11A, B, C, D). Third, in stably transformed Arabidopsis seedlings, the GFP-tagged AtKEA3 was visualized at the typical punctate structures of Golgi bodies spreading within the cells (Figure 11E, F). Thus, the chloroplast localization of AtKEA3 reported by mass spectroscopy might be caused by the contamination with Golgi membranes in the preparation of chloroplasts [47].

The plant Golgi apparatus is an important organelle for polysaccharide synthesis and peptide glycosylation modifications. The Golgi also functions in the trafficking of proteins, lipids, and complex carbohydrates to the cell wall and other organelles [67]. Thus, Golgi localization of AtKEA3 indicates that AtKEA3 may play important roles in the polysaccharide synthesis, peptide modifications, and membrane trafficking processes occurring in the Golgi. This is supported by the result from the hygromycin B assay (Figure 4C), which showed that AtKEA3 conferred resistance to hygromycin B in yeast growth. The Alterations in sensitivity to the cationic drug hygromycin B reflect the changes in membrane potential and membrane trafficking. Indeed, controlling membrane trafficking is an emerging role of Na^+^,K^+^/H^+^ antiporters [8]. It has been shown that yeast ScNhx1p is required for trafficking out of the late endosome [6], [74] [75], and vacuole fusion [57]. Human NHE8 is essential for maintaining endosomal structure and for the regulation of protein sorting [76]. In addition, AtCHX17 and AtCHX21 are involved in protein sorting [33]. *Arabidopsis nhx5nhx6* mutants were defects in vacuolar trafficking [32]. Therefore, AtKEAs may have the same function as the yeast, animal, and plant NHXs and Arabidopsis AtCHX and play important roles in membrane trafficking.

## Supporting Information

Figure S1 **The full-length AtKEA1 is inactive in K+ transport in yeast.**
(TIF)

Figure S2 **The full-length AtKEA1 does not transport K+ at acidic pH in yeast.**
(TIF)

Figure S3 **The full length AtKEA1is not properly distributed in yeast cells.**
(TIF)

Figure S4 **AtKEAs fused with GFP at the C-terminus retained activity.**
(TIF)

Figure S5 **AtKEAs fused with GFP at the C-terminus are properly distributed in yeast cells.**
(TIF)

Materials and Methods S1 **Materials and methods of supporting figures.**
(DOC)

Table S1 **Primers for the plasmid constructs used in functional expression in yeast.**
(DOC)

Table S2 **Primers for RT-qPCR.**
(DOC)

Table S3 **Primers for the plasmid constructs used in localization of GFP fusion proteins in yeast.**
(DOC)
