# Supplementary figures and images for: A Novel AtKEA Gene Family, Homolog of Bacterial K+/H+ Antiporters, Plays Potential Roles in K+ Homeostasis and Osmotic Adjustment in Arabidopsis
Source: PLoS One. 2013 Nov 20;8(11):e81463. doi: 10.1371/journal.pone.0081463 (PMC3835744; doi:10.1371/journal.pone.0081463)

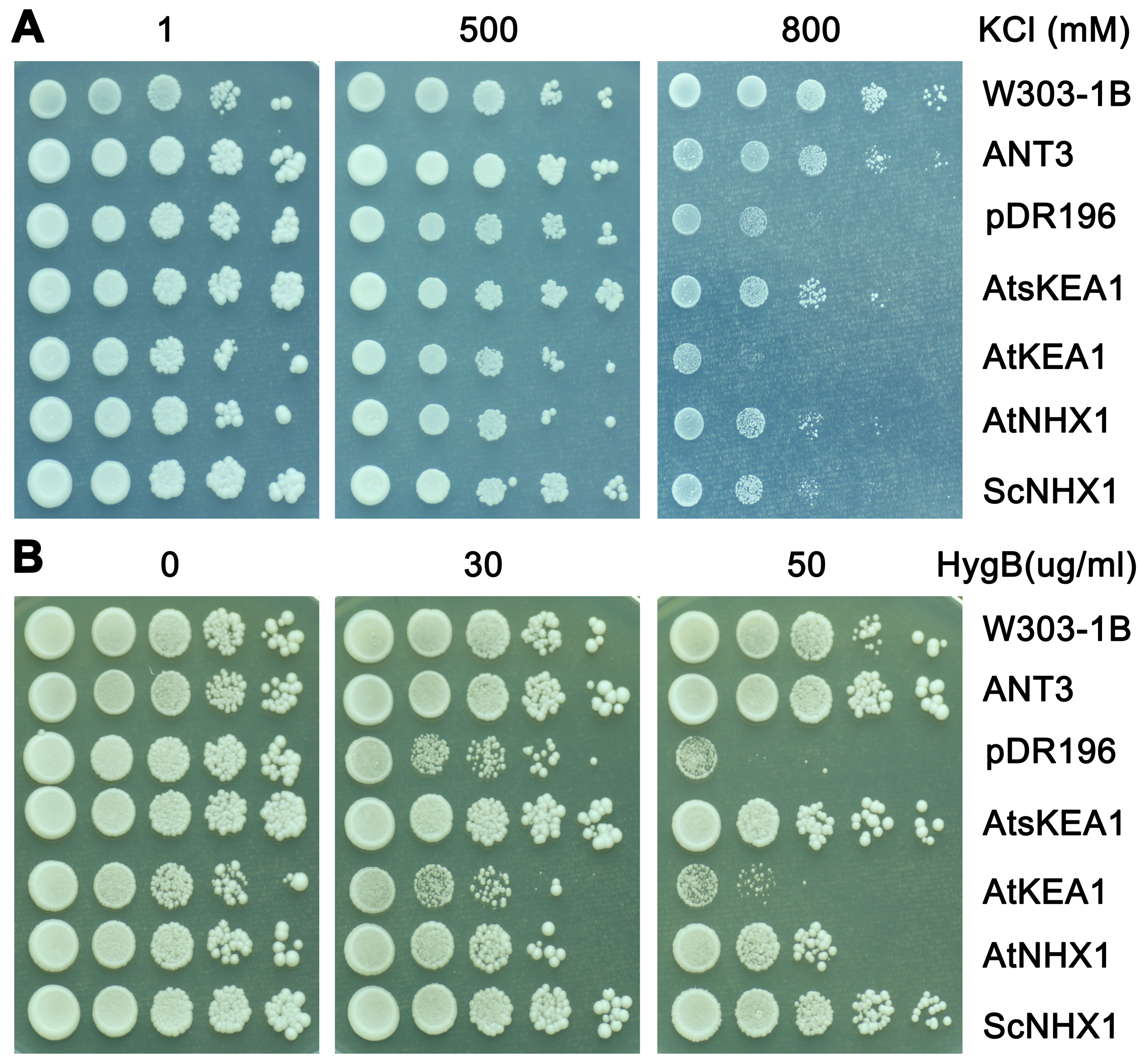

Supplement: Figure S1 — The full-length AtKEA1 is inactive in K+ transport in yeast. (TIF) [file pone.0081463.s001.tif]

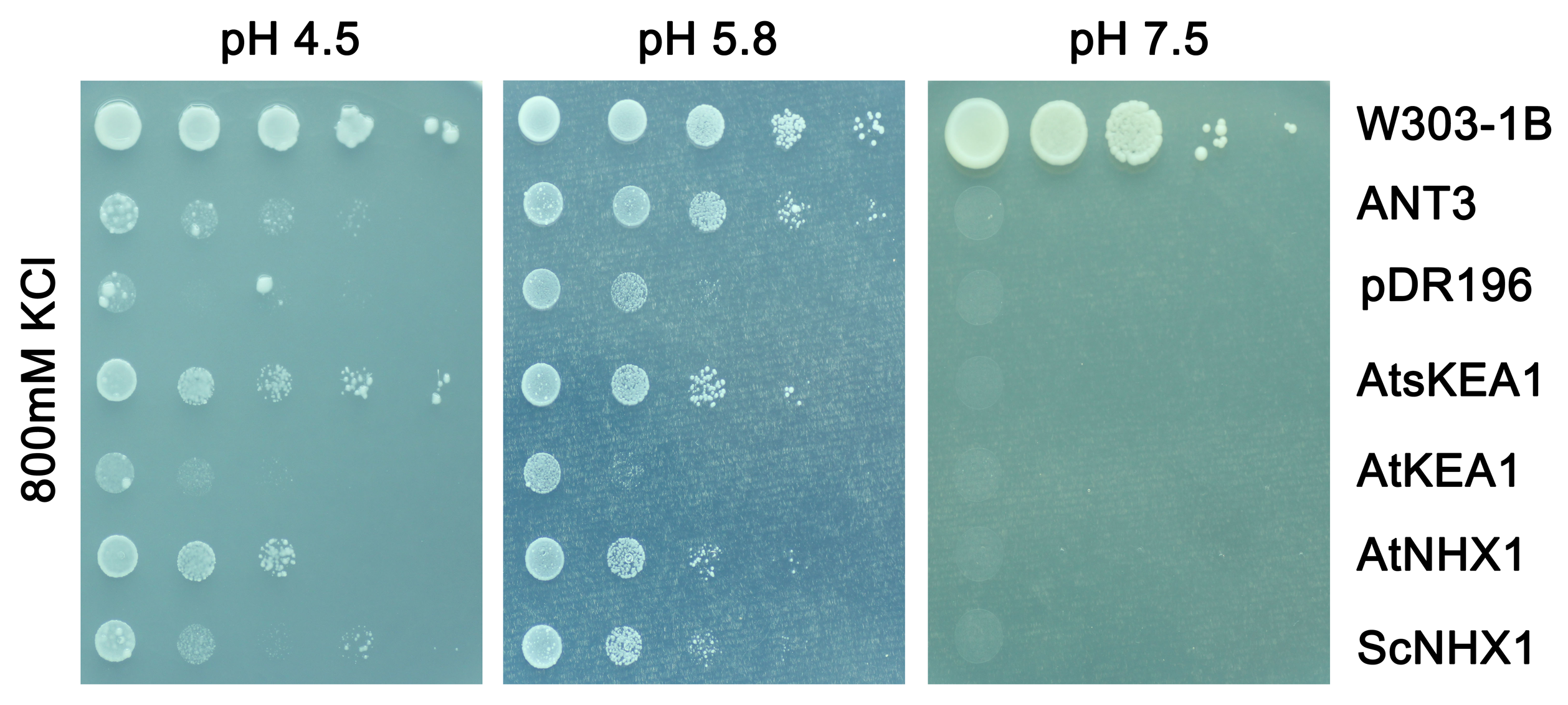

Supplement: Figure S2 — The full-length AtKEA1 does not transport K+ at acidic pH in yeast. (TIF) [file pone.0081463.s002.tif]

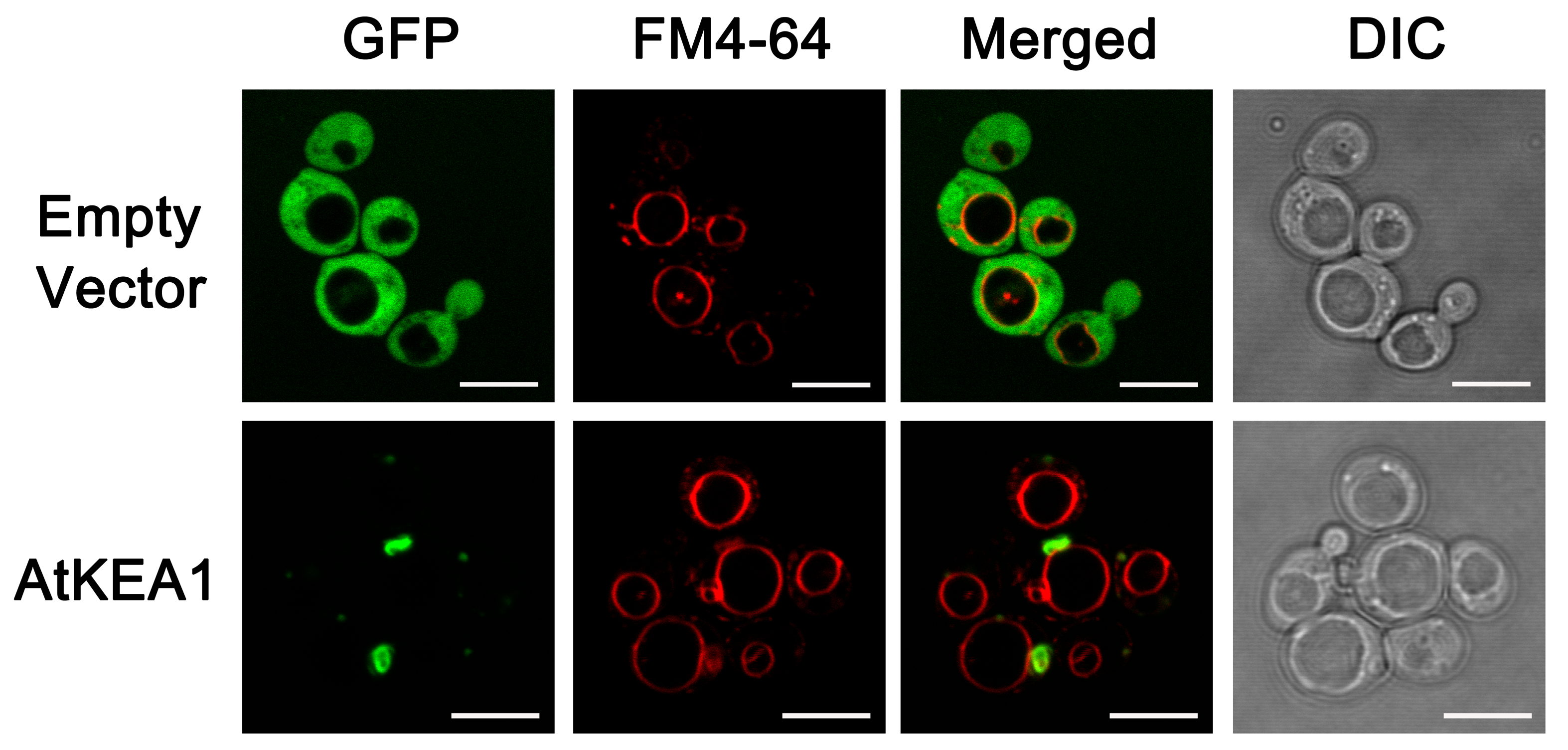

Supplement: Figure S3 — The full length AtKEA1is not properly distributed in yeast cells. (TIF) [file pone.0081463.s003.tif]

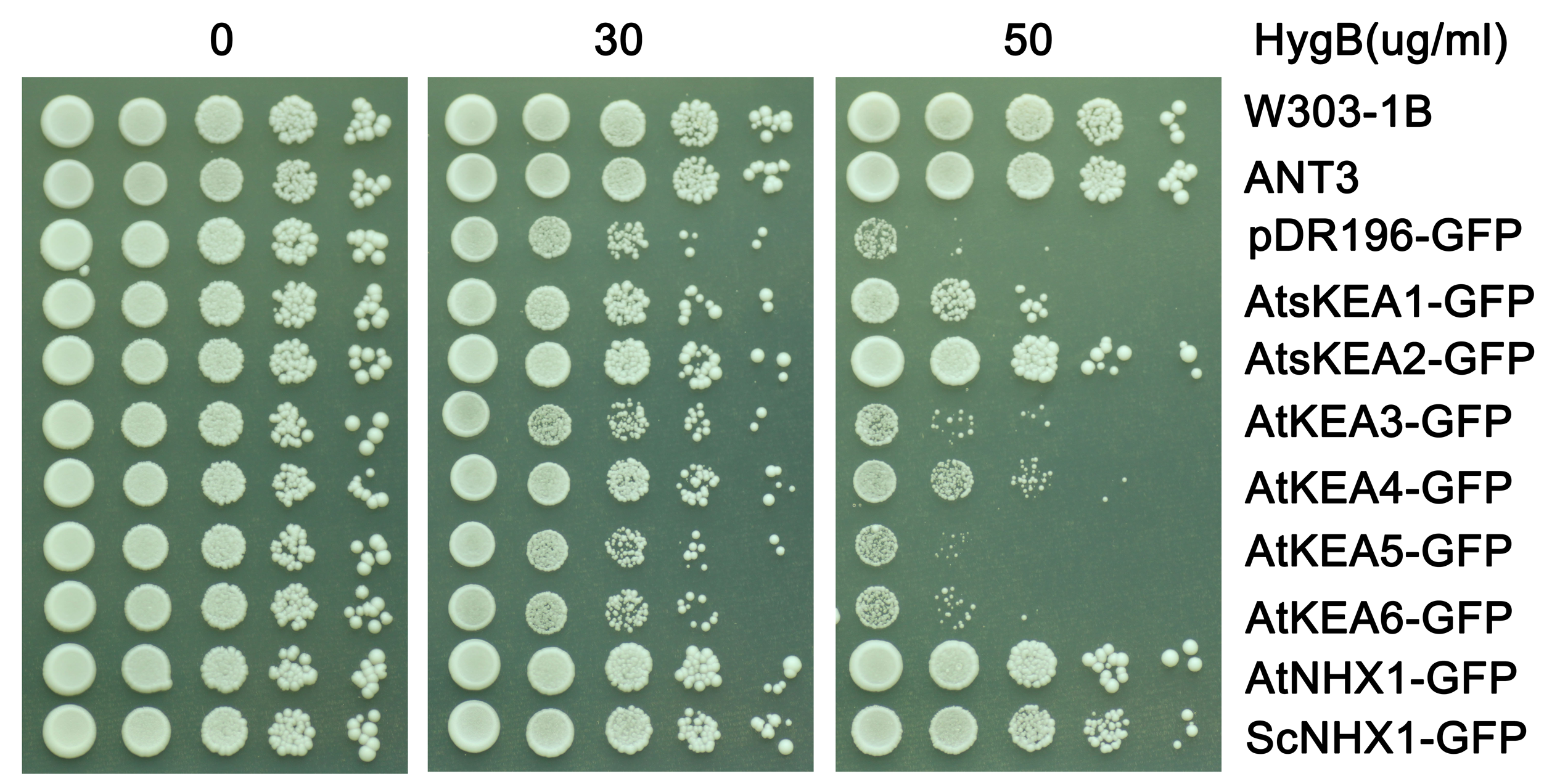

Supplement: Figure S4 — AtKEAs fused with GFP at the C-terminus retained activity. (TIF) [file pone.0081463.s004.tif]

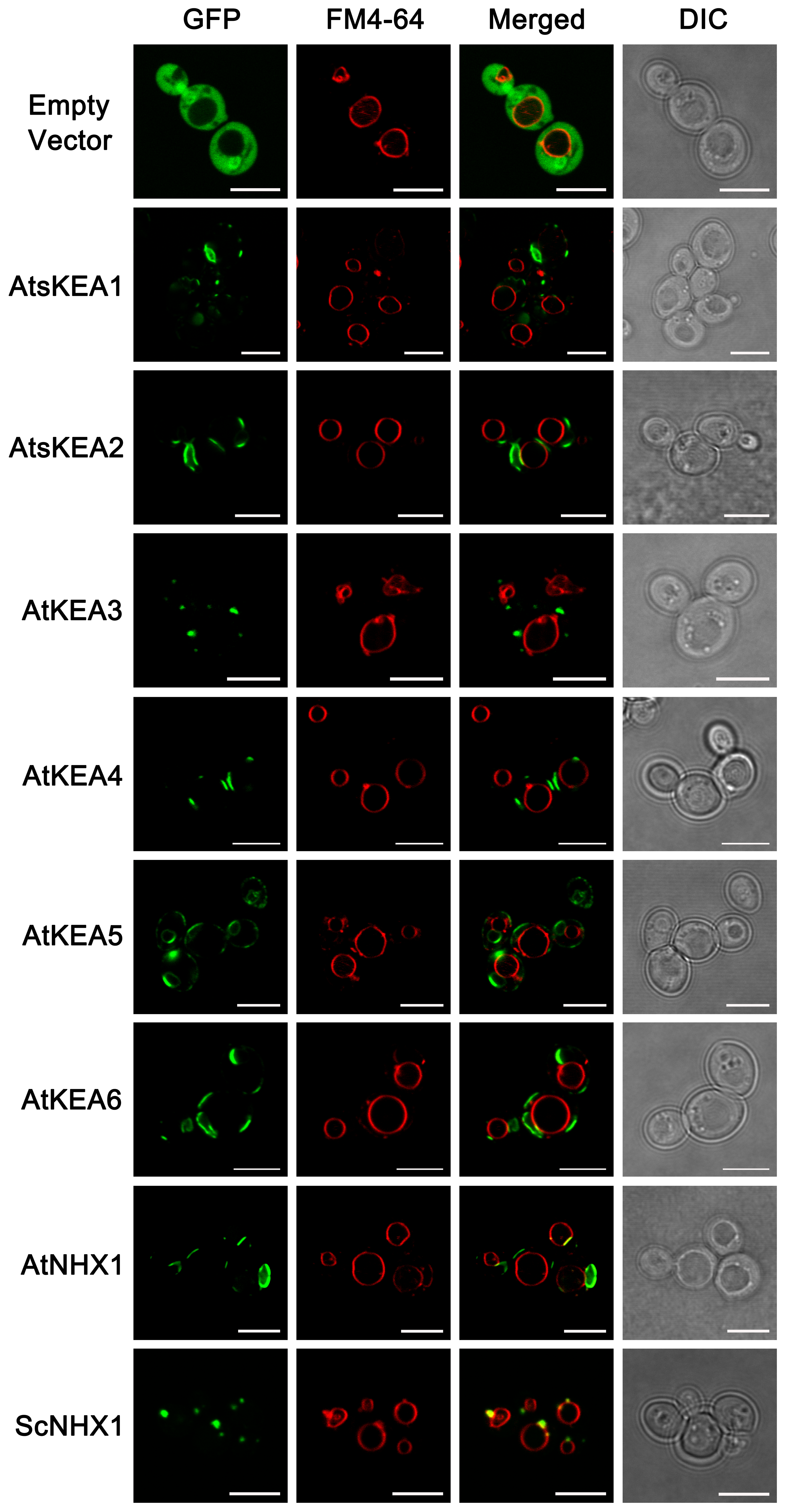

Supplement: Figure S5 — AtKEAs fused with GFP at the C-terminus are properly distributed in yeast cells. (TIF) [file pone.0081463.s005.tif]
