# Supplementary material for: A Novel AtKEA Gene Family, Homolog of Bacterial K+/H+ Antiporters, Plays Potential Roles in K+ Homeostasis and Osmotic Adjustment in Arabidopsis
Source: PLoS One. 2013 Nov 20;8(11):e81463. doi: 10.1371/journal.pone.0081463 (PMC3835744; doi:10.1371/journal.pone.0081463)
Supplement: Materials and Methods S1 — Materials and methods of supporting figures. (DOC) [file pone.0081463.s006.doc]

**Supporting Information**

**Materials and methods**

**Yeast Strains, media, and growth conditions**

*Saccharomyces cerevisiae* strains W303-1B (MATα *leu2-13 112, ura3-1, trp1-1, his3-11 15, ade2-1, can1-100*), ANT3 (*ena1-4Δ::HIS3, nha1Δ::LEU2*), and AXT3 (*ena1-4Δ::HIS3, nha1Δ::LEU2, nhx1Δ:: TRP1*) were gifts from Dr. Jose M. Pardo (Wallis et al., 1989, Quintero et al., 2000, Maresova et al., 2005). All strains used were derivatives of W303-1B. Untransformed strains were grown at 30℃ in YPD medium (1% yeast extract, 2% peptone and 2% glucose). Transformation of yeast cells was performed by the lithium acetate method (Sherman, 2002). After transformation, strains were grown on selective Hartwell's complete (SC) medium or APG medium (10mM arginine, 8mM phosphoric acid, 2 mM MgSO_4_, 1 mM KCl, 0.2 mM CaCl_2_, 2% glucose, and trace minerals and vitamins). KCl, or hygromycin B was added to the medium. Drop test media contained 20 mM MES, and pH was adjusted to 7.5 with arginine (Chanroj et al., 2011) or to acidic pH values with phosphoric acid (Mitsui et al., 2011).

**Functional expression of AtKEAs in yeast**

To clone the full length CDS of AtKEA1, we separated AtKEA1 into two segments, AtKEA1-F1870 and AtKEA1-L1712, respectively, by an EcoRI restriction site in the middle of the gene. Primers KEA1-SmaI F (5′-TATACCCCCGGGATGGAGTATGCGTC-3′) and KEA1-1996R (5′-TGGAACTCAGTCTTTCAACAGATAGCTCAAGGCC-3′) were used to amplify the AtKEA1-F1870 using the PrimeSTAR^TM^ HS DNA Polymerase (TaKaRa) by PCR. AtKEA1-L1712 was amplified with primers KEA1-1862F (5′-CTGGAATTCTGATTGGTCCGT-3′) and KEA1-XhoI R (5′-TAACCGCTCGAGTCAGATTACGACTGTGCCTC-3′) by PCR. The PCR product AtKEA1-F1870 was ligated into the yeast expression vector pDR196 at SmaI-EcoRI sites by T4 DNA ligase (promega), resulting in pDR196-AtKEA1-F1870. Then, the PCR product AtKEA1-L1712 was inserted into the vector pDR196-AtKEA1-F1870 at EcoRI-XhoI sites to obtain the full length AtKEA1 (named as pDR196-AtKEA1). The full length CDS of AtKEA1 was verified by sequencing. To clone AtsKEA1(short form of AtKEA1 with 1857bp nucleotides) and AtNHX1, gene fragments were amplified by PCR from *Arabidopsis* cDNA using the following primers: AtsKEA1 (5′-CGCGTCGACATGATCCCTCACCAGGAGGTC-3′ and 5′-TAACCGCTCGAGTCAGATTACGACTGTGCCTC-3′), AtNHX1 (5′-GGGACTAGTATGTTGGATTCTCTAGTGTC-3′ and 5′-CCGCTCGAGTCAAGCCTTACTAAGATCAG-3′). To clone ScNHX1, the gene fragment was amplified by PCR from the genomic DNA isolated from the *Saccharomyces cerevisiae* strain BJ3505 using the following primers: ScNHX1 (5′-CGCGTCGACATGCTATCCAAGGTATTGC-3′ and 5′-CCGCTCGAGCTAGTGGTTTTGGGAAGAG-3′). The SalI-XhoI PCR fragments of AtsKEA1and ScNHX1, and the SpeI-XhoI PCR fragment of AtNHX1 were cloned into the same sites of the plasmid pDR196, resulting in pDR196-AtsKEA1, pDR196-AtNHX1, and pDR196-ScNHX1, respectively. All gene fragments were verified by sequencing.

All plasmids were transformed into the yeast strain AXT3; the empty vector pDR196 was transformed into the same yeast strains as a control. For stress tolerance tests, yeast cells were normalized in water to A_600_ of 0.4. 4μl aliquots of each 10-fold serial dilution were spotted onto AP plates supplemented with KCl as indicated, and incubated at 30℃ for 3 days. Resistance to hygromycin B was assayed in YPD medium.

**Lcalization of the AtKEAs-GFP fusion proteins in yeast**

To make GFP fusion constructs, we converted the vector pDR196 into a Gateway destination vector pDR196-GFP. GFP was fused at the C-terminal of plant and yeast proteins. Gene fragments of AtKEAs and AtNHX1were amplified by PCR from *Arabidopsis* cDNAs using the following primers: AtsKEA1 (5′-AAAAAGCAGGCTTCATGATCCCTCACCAGGAG-3′ and 5′-AGAAAGCTGGGTCGATTACGACTGTGCCTCC-3′), AtsKEA2 (5′-AAAAAGCAGGCTTCATGTTCCCTCAGCAAGAG-3′ and 5′-AGAAAGCTGGGTCGATAGCGAGTGTGCCTTC-3′), AtKEA3 (5′-AAAAAGCAGGCTTCATGGCAATTAGTACTATGTT-3′ and 5′-AGAAAGCTGGGTCATCTTGAGCTTTATCAGC-3′), AtKEA4 (5′-AAAAAGCAGGCTTCATGCGGCGGTGTAAAAAC-3′ and 5′-AGAAAGCTGGGTCAGAGTCGTGAAGAGAACC-3′), AtKEA5 (5′-AAAAAGCAGGCTTCATGGCGAGATTCGCAGTGATT-3′ and 5′-AGAAAGCTGGGTCCTTGGTTCTGTTATGTACTTCTATCA-3′), AtKEA6 (5′-AAAAAGCAGGCTTCATGGTGGAAGGAAGAAGAAG-3′ and 5′-AGAAAGCTGGGTCGGAGCTGTGGGATTGACG-3′), AtNHX1 (5′- AAAAAGCAGGCTTCATGTTGGATTCTCTAGTGTCG-3′ and 5′- AGAAAGCTGGGTCAGCCTTACTAAGATCAGGAGG-3′). To make the GFP fusion construct for ScNHX1, the gene fragment was amplified by PCR from the genomic DNA isolated from the *Saccharomyces cerevisiae* strain BJ3505 using the following primers: ScNHX1 (5′- AAAAAGCAGGCTTCATGCTATCCAAGGTATTGCTG-3′ and 5′- AGAAAGCTGGGTCGTGGTTTTGGGAAGAGAAAT-3′). The PCR fragment was inserted into the plasmid pDR196-GFP using the Gateway technology (Invitrogen). The gene fragment was verified by sequencing.

To make the GFP fusion construct for AtKEA1, we separated AtKEA1 into two segments at an EcoRI restriction site in the middle of the gene. Primers KEA1-SmaI F (5′-TATACCCCCGGGATGGAGTATGCGTC-3′) and KEA1-1996R (5′-TGGAACTCAGTCTTTCAACAGATAGCTCAAGGCC-3′) were used to amplify the AtKEA1-F1870 using the PrimeSTAR^TM^ HS DNA Polymerase (TaKaRa) by PCR. Then, the PCR product AtKEA1-F1870 was inserted into the vector pDR196-AtsKEA1-GFP at SmaI-EcoRI sites to obtain the full length AtKEA1 (named as pDR196-AtKEA1-GFP). The full length CDS of AtKEA1 was verified by sequencing.

The recombinant plasmids were transformed into the yeast strain AXT3. Yeast cells grown to logarithmic phase at 30℃ in SC-URA medium adjusted to pH 5.8. For FM4-64 staining, yeast cells grown exponentially were harvested and suspended in fresh YPD medium, and then were incubated with FM4-64 dye at a final concentration of 5μM. After incubation for 8h, the cells were washed four times with phosphate buffered saline (PBS) and concentrated by centrifugation. After mixing with 0.6% agarose, the cells were mounted on glass slides and observed by a confocal laser scanning microscope (FV1000, Olympus) (Qiu and Fratti, 2010).

**Figure legends**

**Figure S1. The full-length AtKEA1 is inactive in K^+^ transport in yeast.** The cDNAs of the full length AtKEA1, AtsKEA1, AtNHX1 and ScNHX1were subcloned into the yeast expression vector pDR196 and transformed into strain AXT3 (*ena1-4 nha1 nhx1*). Cells were normalized in water to A_600_ of 0.4. Aliquots (4 μL) from normalized yeast cultures or 10-fold serial dilutions were spotted onto AP plates containing different concentrations of KCl (A), or YPD plates with different concentrations of hygromycin B (B). The strains were grown at 30℃ for 3 days.

**Figure S2. The full-length AtKEA1 does not transport K^+^ at acidic pH in yeast.** The cDNAs of the full length AtKEA1, AtsKEA1, AtNHX1 and ScNHX1 were subcloned into the yeast expression vector pDR196 and transformed into strain AXT3 (*ena1-4 nha1 nhx1*). Strains were spotted onto AP plates containing 800mM KCl at pH 4.5, 5.8 or 7.5. Cells were normalized in water to A_600_ of 0.4. Aliquots (4μL) from normalized yeast cultures or 10-fold serial dilutions were spotted onto AP plates. The strains were grown at 30℃ for 3 days.

**Figure S3. The full length AtKEA1is not properly distributed in yeast cells.** The yeast strain W303-1B harboring the full length AtKEA1 (pDR196-AtKEA1-GFP, GFP was fused at the C terminus) was grown to logarithmic phase in SC-URA medium (pH 5.8) and was stained with FM4-64 dye. The subcellular localization of the GFP-tagged proteins (green) and FM4-64 fluorescence (red) was observed under the Laser Scanning Confocal Microscope. Bars, 5µm.

**Figure S4. AtKEAs fused with GFP at the C-terminus retained activity.** Yeast strains AXT3 harboring pDR196 alone or with AtKEAs, AtNHX1 and ScNHX1 (GFP was fused at the C-terminus) were grown to logarithmic phase in SC-URA medium (pH 5.8). Cells were normalized in water to A_600_ of 0.4. Aliquots (4 µl) from normalized yeast cultures or 10-fold serial dilutions were spotted onto YPD plates containing different concentrations of hygromycin B. The strains were grown at 30℃ for 3 days. AtKEAs fused at the C-terminus with GFP showed the same hygromycin B sensitivity as AtKEAs alone.

**Figure S5. AtKEAs fused with GFP at the C-terminus are properly distributed in yeast cells**. Yeast strains AXT3 (*ena1-4 nha1 nhx1*) harboring pDR196-GFP, pDR196-AtKEAs-GFP, pDR196-AtNHX1-GFP and pDR196-ScNHX1-GFP (fused with GFP at the C terminus) were grown to logarithmic phase in SC-URA medium adjusted (pH 5.8) and were stained with FM4-64 dye. The subcellular localization of the GFP-tagged proteins (green) and FM4-64 fluorescence (red) was observed under the Laser Scanning Confocal Microscope. Bars, 5µm.
