# Supplementary material for: A Novel AtKEA Gene Family, Homolog of Bacterial K+/H+ Antiporters, Plays Potential Roles in K+ Homeostasis and Osmotic Adjustment in Arabidopsis
Source: PLoS One. 2013 Nov 20;8(11):e81463. doi: 10.1371/journal.pone.0081463 (PMC3835744; doi:10.1371/journal.pone.0081463)
Supplement: Table S1 — Primers for the plasmid constructs used in functional expression in yeast. (DOC) [file pone.0081463.s007.doc]

**Table S1. Primers for the plasmid constructs** **used in functional expression in** yeast

| **Primer name** | **Sequence (5′→3′)** |
| --- | --- |
| KEA1-SmaI F | TATACCCCCGGGATGGAGTATGCGTC |
| KEA1-1996 R | TGGAACTCAGTCTTTCAACAGATAGCTCAAGGCC |
| KEA1-1862 F | CTGGAATTCTGATTGGTCCGT |
| KEA1-XhoI R | TAACCGCTCGAGTCAGATTACGACTGTGCCTC |
| KEA2-NotI F | ATAAGAATGCGGCCGCATGGATTTTGCGTCTAG |
| KEA2-2196 R | TGGGATGAGAATCAGTAACACGACCACAGCCAGATC |
| KEA2-2052 F | CAATGGCCTGGCATTGTCTTCCACTGCT |
| KEA2-XbaI R | CGCCGGTCTAGATTAGATAGCGAGTGTGCCTT |
| sKEA1-SalI F | CGCGTCGACATGATCCCTCACCAGGAGGTC |
| sKEA1-XhoI R | TAACCGCTCGAGTCAGATTACGACTGTGCCTC |
| KEA3-SalI F | ACGCGTCGACATGGCAATTAGTACTATGT |
| KEA3-XbaI R | TGCTCTAGATTAATCTTGAGCTTTATCAGCT |
| KEA4-SalI F | CGCGTCGACATGCGGCGGTGTAAAAACAAC |
| KEA4-XhoI R | CCGCTCGAGTCAAGAGTCGTGAAGAGAACC |
| NHX1-SpeI F | GGGACTAGTATGTTGGATTCTCTAGTGTC |
| NHX1-XhoI R | CCGCTCGAGTCAAGCCTTACTAAGATCAG |
| CHX17-PstI F | AAACTGCAGATGGGAACAAACGGTACAAC |
| CHX17-SalI R | CGCGTCGACCTAAGGACTCTCAGAATCC |
| ScNHX1-SalI F | CGCGTCGACATGCTATCCAAGGTATTGC |
| ScNHX1-XhoI R | CCGCTCGAGCTAGTGGTTTTGGGAAGAG |
| ScKHA1-SalI F | CGCGTCGACATGGCAAACACTGTAGGAG |
| ScNHX1-XhoI R | CCGCTCGAGTTATTCAGACGAAAAATGGTG |
