# Supplementary material for: A Novel AtKEA Gene Family, Homolog of Bacterial K+/H+ Antiporters, Plays Potential Roles in K+ Homeostasis and Osmotic Adjustment in Arabidopsis
Source: PLoS One. 2013 Nov 20;8(11):e81463. doi: 10.1371/journal.pone.0081463 (PMC3835744; doi:10.1371/journal.pone.0081463)
Supplement: Table S2 — Primers for RT-qPCR. (DOC) [file pone.0081463.s008.doc]

**Table S2. Primers for** RT-qPCR

| **Primer name** | **Sequence (5′→3′)** |
| --- | --- |
| AtKEA1 F | TCAGGCAAAATTACCGACATC |
| AtKEA1 R | TGGTTGAGGTTTAGGCTTACT |
| AtKEA2 F | TTTTCGGTCTTGCTTTTTCAGG |
| AtKEA2 R | CCAGAAGTGTATTGGCGGAG |
| AtKEA3 F | TCCTGCTCTTTGAGATGGGTC |
| AtKEA3 R | AGAATTTTTGTTCCGATGGCTCC |
| AtKEA4 F | ATGGCGAAATCGTTGGCTAT |
| AtKEA4 R | ACCCAACTCCAGACTTAGAC |
| AtKEA5 F | TAAGCCGAGCGTCAAACCTAC |
| AtKEA5 R | CAATGATGCTTTCTCCTCGTTC |
| AtKEA6 F | TCAGTGAGATGGTCCAGGTTG |
| AtKEA6 R | GCATAACGACACTGTGATACCG |
| AtACTIN7 F | TTCCCGTTCTGCGGTAGTGG |
| AtACTIN7 R | CCGGTATTGTGCTCGATTCTG |
