# Supplementary material for: A Novel AtKEA Gene Family, Homolog of Bacterial K+/H+ Antiporters, Plays Potential Roles in K+ Homeostasis and Osmotic Adjustment in Arabidopsis
Source: PLoS One. 2013 Nov 20;8(11):e81463. doi: 10.1371/journal.pone.0081463 (PMC3835744; doi:10.1371/journal.pone.0081463)
Supplement: Table S3 — Primers for the plasmid constructs used in localization of GFP fusion proteins in yeast. (DOC) [file pone.0081463.s009.doc]

**Table S3. Primers for the plasmid constructs used in localization of GFP fusion proteins in yeast**

| **Primer name** | **Sequence (5′→3′)** |
| --- | --- |
| AtsKEA1 F | AAAAAGCAGGCTTCATGATCCCTCACCAGGAG |
| AtsKEA1 R w/o stop | AGAAAGCTGGGTCGATTACGACTGTGCCTCC |
| AtsKEA2 F | AAAAAGCAGGCTTCATGTTCCCTCAGCAAGAG |
| AtsKEA2 R w/o stop | AGAAAGCTGGGTCGATAGCGAGTGTGCCTTC |
| AtKEA3 F | AAAAAGCAGGCTTCATGGCAATTAGTACTATGTT |
| AtKEA3 R w/o stop | AGAAAGCTGGGTCATCTTGAGCTTTATCAGC |
| AtKEA4 F | AAAAAGCAGGCTTCATGCGGCGGTGTAAAAAC |
| AtKEA4 R w/o stop | AGAAAGCTGGGTCAGAGTCGTGAAGAGAACC |
| AtKEA5 F | AAAAAGCAGGCTTCATGGCGAGATTCGCAGTGATT |
| AtKEA5 R w/o stop | AGAAAGCTGGGTCCTTGGTTCTGTTATGTACTTCTATCA |
| AtKEA6 F | AAAAAGCAGGCTTCATGGTGGAAGGAAGAAGAAG |
| AtKEA6 R w/o stop | AGAAAGCTGGGTCGGAGCTGTGGGATTGACG |
| AtNHX1 F | AAAAAGCAGGCTTCATGTTGGATTCTCTAGTGTCG |
| AtNHX1 R w/o stop | AGAAAGCTGGGTCAGCCTTACTAAGATCAGGAGG |
| AtCHX17 F | AAAAAGCAGGCTTCATGGGAACAAACGGTACAAC |
| AtCHX17 R w/o stop | AGAAAGCTGGGTCAGGACTCTCAGAATCCTCAAC |
| ScNHX1 F | AAAAAGCAGGCTTCATGCTATCCAAGGTATTGCTG |
| ScNHX1 R w/o stop | AGAAAGCTGGGTCGTGGTTTTGGGAAGAGAAAT |
| ScKHA1 F | AAAAAGCAGGCTTCATGGCAAACACTGTAGGAGGA |
| ScKHA1 R w/o stop | AGAAAGCTGGGTCTTCAGACGAAAAATGGTGCA |
